# Meeting 24-hour movement guidelines among Canadian post-secondary students: temporal trends, disparities, and mental health associations

**DOI:** 10.1186/s12966-026-01952-7

**Published:** 2026-07-16

**Authors:** Meijun Hou, Mark R. Beauchamp, Matthew Y. W. Kwan, Guy Faulkner

**Affiliations:** 1 School of Kinesiology, The University of British Columbia, Vancouver, V6T 1Z1 Canada; 2 Department of Child and Youth Studies, Brock University, St. Catharines, L2S 3A1 Canada; 3 Department of Community Health Sciences, University of Calgary, Calgary, T2N 4N1 Canada; 4 Health and Wellbeing Center for Research Innovation, School of Human Movement and Nutrition Sciences, The University of Queensland, Brisbane, 4072 Australia; 5 School of Kinesiology, The University of British Columbia, Gateway Health Building, 5955 University Blvd, Vancouver, V6T 1Z1 Canada

**Keywords:** Young adults, Movement behaviors, Psychological factors, Health disparities

## Abstract

**Background:**

Meeting the 24-Hour Movement Guidelines (24-HMG), comprising aerobic physical activity, sedentary behavior including recreational screen time, and sleep, is critical for post-secondary student health. However, multi-year temporal trends spanning the pandemic and post-pandemic period, as well as sociodemographic and mental health disparities, remain understudied in Canada. This limits the development of targeted interventions.

**Methods:**

We conducted a serial cross-sectional analysis using Canadian Campus Wellbeing Survey data collected between 2019 and 2025. A self-reported questionnaire was administered annually to assess aerobic physical activity, sedentary behavior (including recreational screen time), sleep duration, sociodemographic characteristics, and mental health. Prevalence of meeting individual and integrated 24-HMG components was estimated. Temporal trends were examined using regression models with survey year treated as a continuous variable and were further stratified by sociodemographic characteristics and mental health status. Multivariable logistic regression was used to examine factors associated with meeting all guidelines and meeting none.

**Results:**

Among 116,597 students (mean [SD] age, 23.95[8.02] years; 78,623[67.43%] women), divergent trends emerged. From 2019 to 2025, meeting physical activity and sedentary behavior guidelines increased, whereas meeting recreational screen time and sleep guidelines declined. The proportion meeting none of the guidelines significantly decreased, ranging from 7.45% to 4.44% (-0.29% [-0.30, -0.22], *p* < 0.001). Meeting all guidelines (14.66% in 2019, 8.92% in 2021, 11.74% in 2025) showed a positive linear trend (*β* = 0.30% [95% CI: 0.20, 0.40], *p* < 0.001) that sensitivity analyses attributed to transient COVID-19 fluctuations. Meeting none of the guidelines significantly decreased (*β* = -0.29% [-0.30, -0.22], *p* < 0.001). Disparities were observed: younger students (≤ 24 years), those identifying as non-binary/Two-Spirit or Black, those reporting lower parental education or high financial stress, and those experiencing severe mental distress or low well-being were less likely to meet integrated guidelines.

**Conclusions:**

While post-pandemic improvements were observed in physical activity and sedentary time, prevalence of meeting recreational screen time and sleep guidelines declined over time. Overall compliance with all movement components remains low and socially patterned. Continued surveillance and focused, equity-informed campus health strategies are warranted to address persistent behavioral and mental health disparities in the digital era.

**Supplementary Information:**

The online version contains supplementary material available at https://doi.org/10.1186/s12966-026-01952-7.

## Background

Post-secondary education is a critical developmental transition often accompanied by academic and financial stress, shifting social environments, which is viewed through the lens of life course health development [1–3]. Emerging adulthood is also a period of elevated vulnerability to mental health challenges [4, 5], with more than 75% of mental disorders having onset before age 25 [6]. Recent evidence indicates rising prevalence of mental health concerns among university students [7–9]. Although much of this literature has focused on traditional-aged undergraduates (e.g., 18–24), the burden of mental disorders is not confined to early adulthood. Prevalence shows a roughly linear increase extending into late middle age, with median ages of onset spanning the late 20s to early 40s [7], suggesting that students across a broad age range remain at meaningful risk. During this critical period, when long-term lifestyle habits are established [10], movement behavior patterns including physical activity, sedentary behavior, and sleep may play an important role in supporting physical and psychological health [11, 12]. Research on movement behaviors in post-secondary populations has predominantly focused on traditional-aged undergraduates, leaving more age-diverse student populations understudied. Heavy screen use and prolonged sedentary study time are also standard in contemporary student life, highlighting the need for population-level surveillance [13, 14].

The Canadian 24-Hour Movement Guidelines (24-HMG) for adults (18–64 years) provide an integrated framework for daily time use, emphasizing moderate-to-vigorous aerobic physical activity (MVPA: ≥150 min/week), sedentary behavior (SB; as ≤ 8 h/day with ≤ 3 h of recreational screen time [ST]), and sleep (7–9 h/night) [15]. Unlike single-behavior recommendations, the 24-HMG conceptualize movement behaviors as integrated components within a finite 24-hour period. Evidence has consistently shown associations between meeting the 24-HMG and reduced mortality and improved mental health [16, 17]. Within this framework, a critical distinction is made between recreational screen time and overall sedentary time: several studies have demonstrated that recreational screen-based activities are independently associated with anxiety and depressive symptoms, distinct from non-screen sedentary tasks like academic work [18–20], suggesting that examining recreational screen time separately may better clarify its role within the broader sedentary behaviors spectrum.

Over the past decade, digital media use has become increasingly embedded in young adults’ academic and social lives [21, 22]. The COVID-19 pandemic further accelerated this shift through remote learning, reduced in-person activity, and expanded online engagement [23]. Screen time increased substantially among university students during the pandemic, often coinciding with changes in PA and sleep [24]. Although public health restrictions have largely subsided [25], it remains unclear whether these behavior changes have persisted or whether students have returned to pre-pandemic patterns.

Despite the relevance of integrated movement behaviors for young adult health, continuous multi-year surveillance of meeting 24-HMG among post-secondary students remains fragmented. National monitoring systems often aggregate young adults into the general adult population (18–64 years), potentially obscuring the unique behavioral patterns of students [26, 27]. Few studies have examined trends spanning pandemic and post-pandemic periods or explored how sociodemographic and mental health characteristics are associated with meeting integrated movement guidelines over time. Existing surveillance has often focused on individual behaviors or younger age groups [12, 20, 28], and although sociodemographic factors (e.g., gender, race/ethnicity, financial stress) and mental health status are known to differentially influence movement behaviors [29, 30], little research has examined how these characteristics shape adherence to integrated movement guidelines within post-secondary populations. These disparities in health-promoting behaviors may contribute to broader health inequities [31–33].

The Canadian Campus Wellbeing Survey (CCWS), a large repeated cross-sectional survey conducted across Canadian post-secondary institutions since 2019, includes standardized measures of 24-HMG and mental health [34], offering an ideal dataset to address these gaps. Although movement behaviors among Canadian young adults have been examined previously [35–37], robust multi-year trends in meeting integrated 24-HMG remain limited, and little is known about whether sociodemographic and mental health correlates of meeting these guidelines have shifted over time. This study therefore aimed to: (1) examine 7-year trends (2019–2025) in the prevalence of meeting individual and integrated 24-HMG components; (2) compare patterns between pandemic-affected and post-pandemic periods; and (3) examine disparities in meeting the integrated guidelines across sociodemographic subgroups and mental health status. We hypothesized that meeting all guidelines would be associated with lower mental distress and higher mental well-being, while meeting none would show the opposite pattern.

## Methods

### Study design and participants

The CCWS monitors student health and well-being using an institutional opt-in approach, followed by census or random sampling within participating Canadian post-secondary institutions; students are invited via email with a unique online link. Data were collected across 10 distinct waves: Fall 2019, Winter and Fall 2020, Winter and Fall 2021, Winter 2022, Winter and Fall 2023, Winter 2024, and Winter 2025. Survey deployment periods occurred during regular Fall and Winter academic terms, with data collection spanning from September through June. The Winter 2020 survey deployment period overlapped with the onset of the COVID-19 pandemic in Canada, during which many post-secondary institutions transitioned to remote instruction and implemented restrictions on access to campus facilities, including recreational and athletic spaces. The average response rate was 14.9% (range: 11.8% − 18.8%). Further design details are available at https://www.ccws-becc.ca/.

The final pooled dataset included 147,061 participants aged 18–64 from 97 unique institutions (1 participated in 6 waves, 1 in 4, 10 in 3, 29 in 2, and 56 in a single wave). This dataset merged information on 24-HMG, sociodemographic, and mental health characteristics among students. After excluding 30,464, students due to incomplete responses., the final analytic sample comprised 116,597. This serial cross-sectional study received approval from the first author’s Behavioural Research Ethics Board and the CCWS Data Access Committee.

### Assessment of 24-hour movement behaviors

Participants were classified as meeting or not meeting each 24-HMG domain [15] for aerobic physical activity (PA), sedentary behavior (SB), recreational screen time (ST), and sleep based on self-reported data (Supplement eTable 1).

#### Aerobic PA

MVPA was assessed using the International Physical Activity Questionnaire (IPAQ) short-form items [38]. Participants reported days/week and minutes/day of moderate and vigorous PA. The measure does not differentiate activity domains (e.g., leisure, occupational, transport). Following standard IPAQ truncation protocols, daily PA exceeding 180 min was truncated. Participants achieving ≥ 150 min/week of MVPA were classified as meeting the PA guideline. The IPAQ Short Form has demonstrated moderate test-retest reliability (ICC = 0.52) and acceptable concurrent validity in adult and university populations [38].

#### SB and recreational ST

Two components were assessed independently: (i) Total SB: Participants estimated total daily sitting time over the last 7 days. Participants reporting ≤ 8 h/day were classified as meeting the SB guideline. (ii) Recreational ST: Recreational ST was assessed using items derived from the International Sedentary Assessment Tool (ISAT; α = 0.80) [39], capturing time spent watching TV, playing video games, or using the internet for non-academic/non-work purposes on a typical day. Participants reporting no more than 3 h/day of recreational ST were classified as meeting the guideline [40].

#### Sleep duration

Sleep was derived from four questions regarding usual bedtimes and wake-up times on both weekdays and weekends adhering to recommendations for sleep health surveillance in Canada [41]. Average daily sleep duration was calculated based on the difference between reported bed and wake times. 7–9 h were classified as meeting the guideline. Total sedentary behavior and sleep duration were assessed using single-item measures commonly used in adult and post-secondary populations for large-scale surveillance [42, 43]. Composite variables identified participants meeting all guidelines and those meeting none [44, 45].

### Assessment of sociodemographic and psychological characteristics

Sociodemographic data including age, gender (women, men, and non-binary/Two-Spirit), race/ethnicity (White, Black, Asian, Multiracial, and Other), and parental educational level (high school or less, college/technical, university degree, and graduate or higher). Age was reported as a continuous variable in descriptive statistics and modeled as a five-category variable (< 20, 20–24, 25–29, 30–34, ≥ 35) in stratified and multivariable analyses to allow for non-linear effects across life-stage transitions in the post-secondary population. Financial stress was assessed on a 5-point scale ranging from “no financial stress at all” to “a great deal of financial stress”. Mental distress was assessed using the Kessler Psychological Distress Scale (K10) [46], categorized as no/little, mild, moderate, and severe, with acceptable psychometric properties across sociodemographic subgroups [47]. Mental well-being was assessed using the Warwick-Edinburgh mental well-being scale (WEMWBS), which has shown high test-retest reliability [48]. Scores were categorized as low, average, and high.

### Statistical analysis

All analyses were conducted using Stata version 17.0 (StataCorp, College Station, TX, USA). Descriptive statistics used means and standard deviations (*SD*) for continuous variables and frequencies and percentages for categorical variables. Differences between included and excluded participants were assessed using t-tests and *Chi*-square. Unadjusted linear regression models estimated temporal trends in meeting 24-HMG, with survey year as a continuous predictor. Results are reported as unstandardized coefficients (*β*) with 95% confidence intervals (CIs), representing average annual percentage-point changes. Trend analyses were stratified by age, gender, ethnicity, parental educational level, financial stress, and mental health. Pandemic/transition period (2020–2022) and post-pandemic recovery period (2023–2025) were compared using logistic regression models (2019 data excluded), with results presented as ORs with 95% CIs.

Multivariable logistic regression examined sociodemographic and mental health correlates of meeting all versus none of the guidelines, adjusted for age, gender, ethnicity, parental educational level, financial stress, mental distress, mental well-being, and survey year. Results are presented as adjusted Odds Ratios (aOR) with 95%CIs. Multicollinearity was assessed using the Variance Inflation Factor (VIF), the mean VIF was 1.58, well below the threshold of 5.

Sensitivity analyses were conducted to evaluate robustness of findings. First, trend analyses were repeated excluding peak pandemic years (2020–2021) to assess the underlying secular trends free from acute global restrictions. Second, temporal trend models were additionally adjusted for sociodemographic and mental health covariates to assess whether the observed trends were influenced by changes in sample composition over time. All statistical tests were 2-sided with statistical significance set at *p* < 0.05. *p* values were not adjusted for multiple testing and should be interpreted as exploratory.

## Results

### Participant characteristics

Of 147,061 respondents, 30,464 (20.7%) were excluded due to missing data. Comparisons between the included and excluded samples are presented in Supplementary eTable 2. While the sociodemographic structure remained largely comparable (e.g., gender distribution), excluded participants were more likely to identify as Asian and report severe mental distress. However, exclusion rates remained stable across survey years (17.0% − 23.9%). Sensitivity analyses adjusting for these factors yielded temporal trends virtually identical to the unadjusted estimates (difference < 1%), confirming that the observed patterns were not driven by attrition bias. Compositional shifts in sociodemographic distributions were observed across the seven survey cycles (Supplementary eFigure. 1). The final analytic sample (*N* = 116,597) had a mean age of 23.95 years (*SD* = 8.02), with the majority identifying as women (67.43%, *n* = 78,623) and as White (48.37%, *n* = 56,397). Detailed characteristics are in Table 1. The annual sample sizes and distributions are in Supplement eTable 3.

**Table 1:** Characteristics of Canadian post-secondary students, CCWS 2019–2025 (*N* = 116,597)

	Mean / *N*^a^	SD / %^a^
Age (continuous)	23.95	8.02
Age group		
Under 20	31,510	27.02
20–24	50,990	43.73
25–29	15,786	13.54
30–34	7,755	6.65
Over 35	10,556	9.05
Gender		
Women	78,623	67.43
Men	34,862	29.90
Non-binary/Two-spirit	3,112	2.67
Ethnicity		
White	56,397	48.37
Black	4,613	3.96
Asian	39,848	34.18
Multiracial	7,417	6.36
Other	8,322	7.14
Parental educational level		
High school or less	23,479	20.14
College or Technical	24,890	21.35
University degree	40,574	34.80
Graduate or higher	27,654	23.72
Financial stress		
No financial stress at all	14,005	12.01
Very little financial stress	23,601	20.24
Some financial stress	34,706	29.77
Quite a bit of financial stress	23,023	19.75
A great deal of financial stress	21,262	18.24
Mental distress		
Little/no mental distress	27,532	23.61
Mild mental distress	24,184	20.74
Moderate mental distress	24,046	20.62
Severe mental distress	40,835	35.02
Mental well-being		
Low mental well-being	38,370	32.91
Average mental well-being	68,898	59.09
High mental well-being	9,329	8.00

^a^ *N* represent sample counts and % is percentage of sample sizes

Across the seven-year period, average prevalence of meeting individual guidelines was 66.96% for PA, 54.52% for SB, 35.97% for recreational ST, and 61.91% for sleep. Only 11.39% of participants met all guidelines, while 5.64% met none (Table 2; Supplement eTable 4).

**Table 2:** Prevalence and trends in meeting 24-hour movement guidelines among Canadian post-secondary students, CCWS 2019–2025

Movement Behavior	2019	2020	2021	2022	2023	2024	2025	7-year Linear Trend	Pandemic Comparison
								2019–2025	(Post vs. During)
	%	%	%	%	%	%	%	β (95%CI)	OR (95% CI) ^a^
Aerobic PA	67.74	64.53	63.04	63.94	68.03	70.96	68.87	0.01 (0.009, 0.011)***	1.28 (1.25, 1.31) ***
SB	58.10	53.53	41.29	49.89	60.12	60.07	59.06	0.02 (0.018, 0.021)***	1.56 (1.52, 1.60) ***
Recreational ST	43.46	36.09	35.09	33.32	38.39	37.47	34.26	-0.004 (-0.006, -0.003)***	1.08 (1.05, 1.11) ***
Sleep	62.63	61.58	64.23	62.11	60.92	62.18	60.43	-0.003 (-0.004, -0.001)***	0.95 (0.93, 0.97) ***
Meeting all	14.66	11.08	8.92	9.62	12.66	13.12	11.74	0.003 (0.002, 0.003) ***	1.30 (1.25, 1.35) ***
Meeting none	4.44	5.99	7.45	6.62	4.69	4.47	5.22	-0.003 (-0.003, -0.002)***	0.71 (0.67, 0.74) ***

*Abbreviations: PA* physical activity, *SB* sedentary behavior, *ST* screen time

^a^ Pandemic Comparison: Odds Ratios (OR) were calculated using logistic regression comparing the post-pandemic period (2023–2025) to the During-pandemic period (2020–2022, as reference group). An OR > 1 indicates a higher likelihood of meeting guidelines in the post-pandemic period

*** *P* < 0.001

### Temporal trends in meeting 24-HMG

**Fig. 1:**
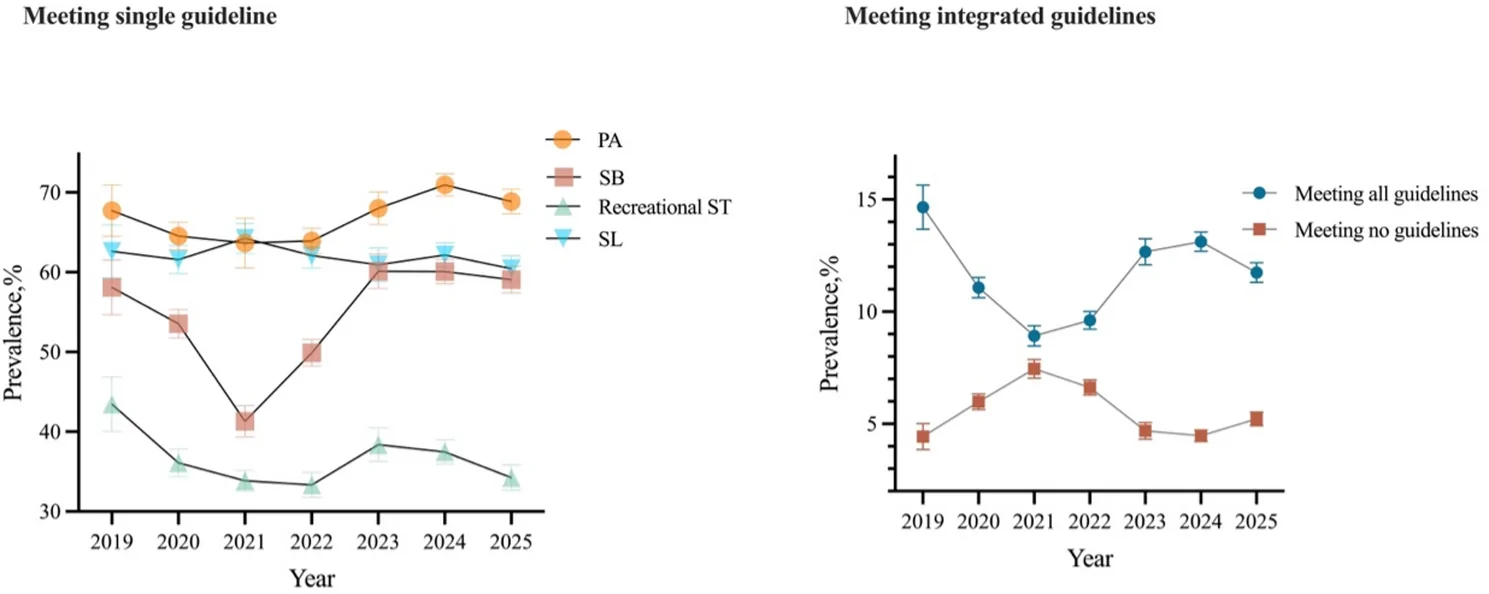
Crude seven-year trends in meeting single and integrated 24-HMG among Canadian post-secondary students, CCWS 2019–2025. Abbreviations: PA = aerobic physical activity; SB = sedentary behavior; Recreational ST = recreational screen time; SL = sleep duration

Figure 1 illustrates seven-year trends in meeting individual and integrated guidelines (Table 2). PA adherence remained the highest throughout, dipping from 67.74% in 2019 to 63.04% in 2021 before recovering to 68.87% by 2025 (*β* = 0.01, *p* < 0.001). Adherence to SB guidelines followed a steeper trajectory, falling sharply from 58.10% in 2019 to 41.29% in 2021 and rebounding to 59.06% by 2025 (*β* = 0.02, *p* < 0.001). In contrast, recreational ST adherence declined steadily from 43.46% in 2019 to 34.26% in 2025 (*β* = -0.004, *p* < 0.001), and sleep adherence declined modestly from 62.63% to 60.43% (*β* = -0.003, *p* < 0.001). Meeting all guidelines fell from 14.66% in 2019 to 8.92% in 2021 before partially recovering to 11.74% by 2025 (*β* = 0.003, *p* < 0.001), while meeting none rose from 4.44% in 2019 to a pandemic peak of 7.45% in 2021 before returning toward baseline (5.22% in 2025; *β* = -0.003, *p* < 0.001).

Compared to the pandemic period, post-pandemic participants had higher odds of meeting PA (OR = 1.28, 95% CI 1.25, 1.31), SB (OR = 1.56, 95% CI 1.52, 1.60), and recreational ST guidelines (OR = 1.08, 95% CI 1.05, 1.11; all *p* < 0.001), but lower odds of meeting the sleep guideline (OR = 0.95, 95% CI 0.93, 0.97, *p* < 0.001). They were also more likely to meet all guidelines (OR = 1.30, 95% CI 1.25, 1.35) and less likely to meet none (OR = 0.71, 95% CI 0.67, 0.74; both *p* < 0.001). Sensitivity analyses excluding the peak pandemic years (2020–2021) confirmed robust long-term trends for individual behaviors and meeting no guidelines (*p* < 0.05). However, the trend for meeting all guidelines became non-significant (*p* = 0.78), suggesting that the observed fluctuations in full adherence were primarily driven by pandemic-related anomalies rather than a consistent linear trajectory, as the positive trend did not persist after excluding the peak pandemic years (Supplement eTable 5).

### Temporal trends in 24-HMG across sociodemographic and mental health characteristics

**Fig. 2:**
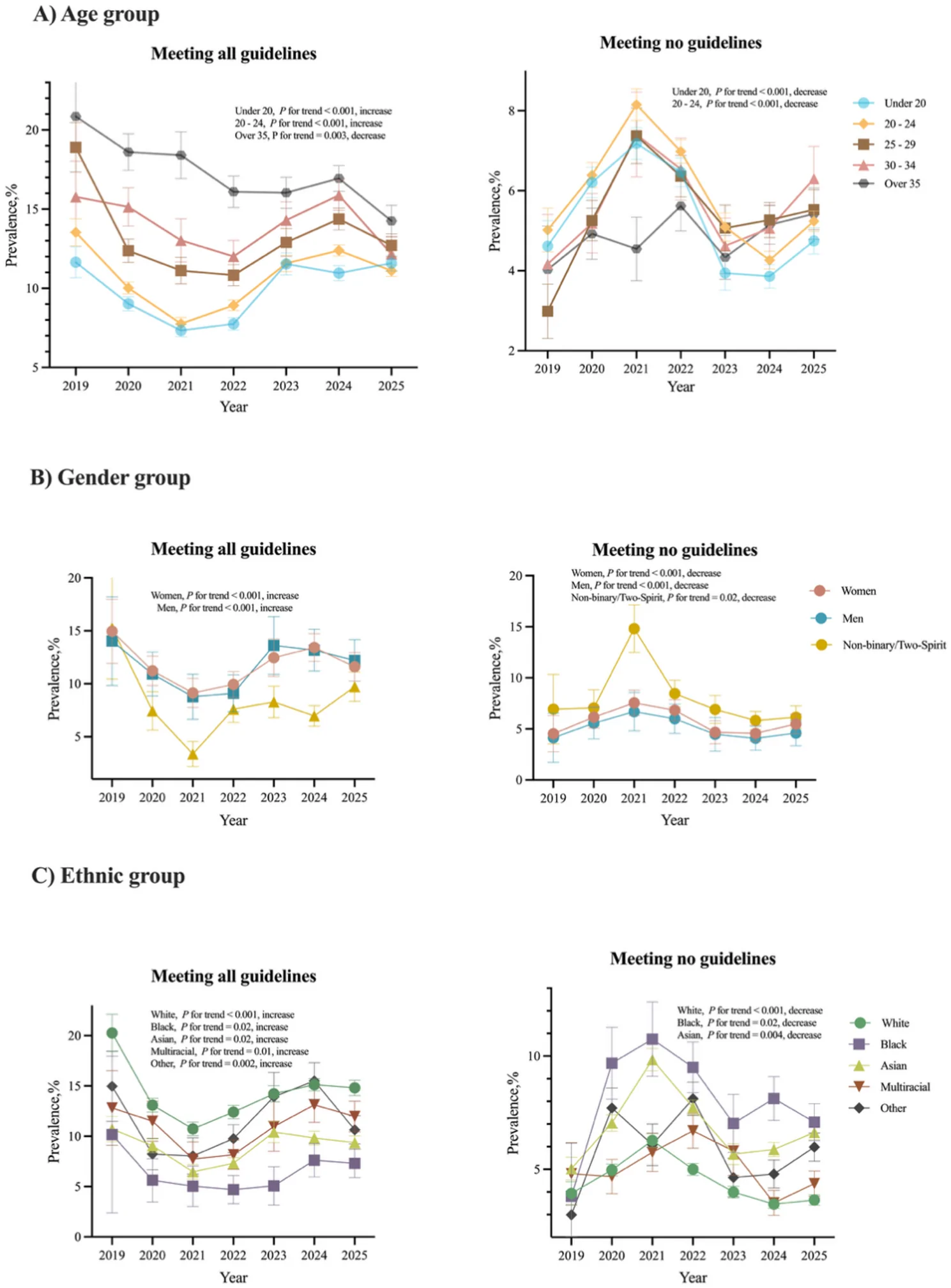
Trends in the prevalence of meeting 24-hour movement guidelines stratified by demographic characteristics, CCWS 2019–2025. **A** Age group. **B **Gender group. **C **Ethnic group

**Fig. 3:**
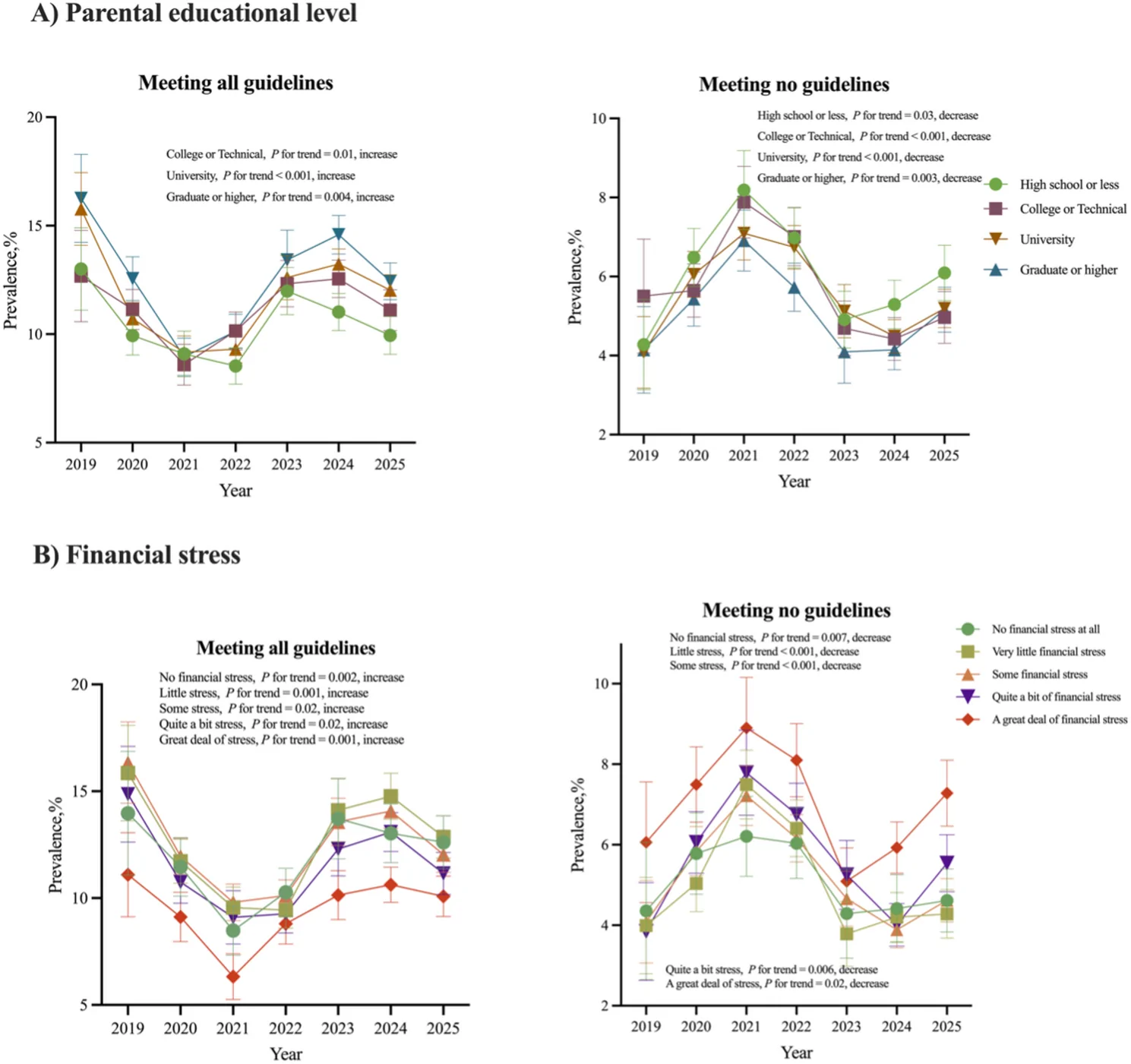
Trends in the prevalence of meeting 24-hour movement guidelines stratified by socioeconomic characteristics, CCWS 2019–2025. **A** Parental education level. **B** Financial stress

The temporal trends in meeting the 24-HMG stratified by sociodemographic characteristics are presented in Figs. 2 and 3, with further prevalence and trend details in Supplementary eTables 6–12.

#### Age

Meeting all guidelines significantly increased over time among students aged ≤ 24 years (both *p* < 0.001), accompanied by significant decreases in meeting none (both *p* < 0.001). In contrast, students aged ≥ 35 years showed a significant decline in meeting all guidelines, falling from 20.9% in 2019 to 14.3% in 2025 (*p* < 0.01), despite consistently having the highest baseline prevalence across the period. Trends among students aged 25–29 and 30–34 were not statistically significant.

#### Gender

Both women and men demonstrated significant increasing trends in meeting all guidelines and significant decreasing trends in meeting none (both *p* < 0.001). Non-binary/Two-Spirit participants reported the lowest prevalence of meeting all guidelines and the highest prevalence of meeting none across the period, with pronounced pandemic-related disruption: meeting all dropped from 15.3% in 2019 to a low of 3.4% in 2021 before partially recovering to 9.7% in 2025, while meeting none peaked at 14.8% in 2021 before declining to 6.1% by 2025 (*p* < 0.01).

#### Ethnicity

White participants reported the highest prevalence of meeting all guidelines across the period (from 20.3% in 2019 to 14.8% in 2025) and the lowest prevalence of meeting none, while Black participants reported the lowest prevalence of meeting all (from 10.2% in 2019 to 7.3% in 2025) and the highest prevalence of meeting none, peaking during the pandemic (10.8% in 2021). Despite these disparities, all ethnic groups showed significant increasing trends in meeting all guidelines over the seven-year period (all *p* < 0.05), and significant decreases in meeting none were observed among White (*p* < 0.001), Black (*p* = 0.02), and Asian (*p* = 0.004) participants. Stratified estimates for individual 24-HMG components are reported in eTables 6–8.

#### Parental educational level

A significant decreasing trend was observed in meeting none of the guidelines (*p* = 0.02). Significant increasing trends in meeting all guidelines were observed among participants whose parents had a college/technical (*p* = 0.01), university (*p* < 0.001), or graduate degree or higher (*p* = 0.001), accompanied by significant decreasing trends in meeting none across these three groups (all *p* ≤ 0.001).

#### Financial stress

Participants experiencing a great deal of financial stress consistently reported the lowest prevalence of meeting all guidelines, falling from 11.1% in 2019 to a low of 6.3% in 2021 before partially recovering to 10.0% by 2025, and the highest prevalence of meeting none, peaking at 8.9% in 2021 before declining to 7.3% by 2025. Despite these disparities, significant increasing trends in meeting all guidelines and decreasing trends in meeting none were observed across all financial stress categories (all *p* < 0.05). Stratified estimates for individual 24-HMG components are reported in eTables 9 and 10.

**Fig. 4:**
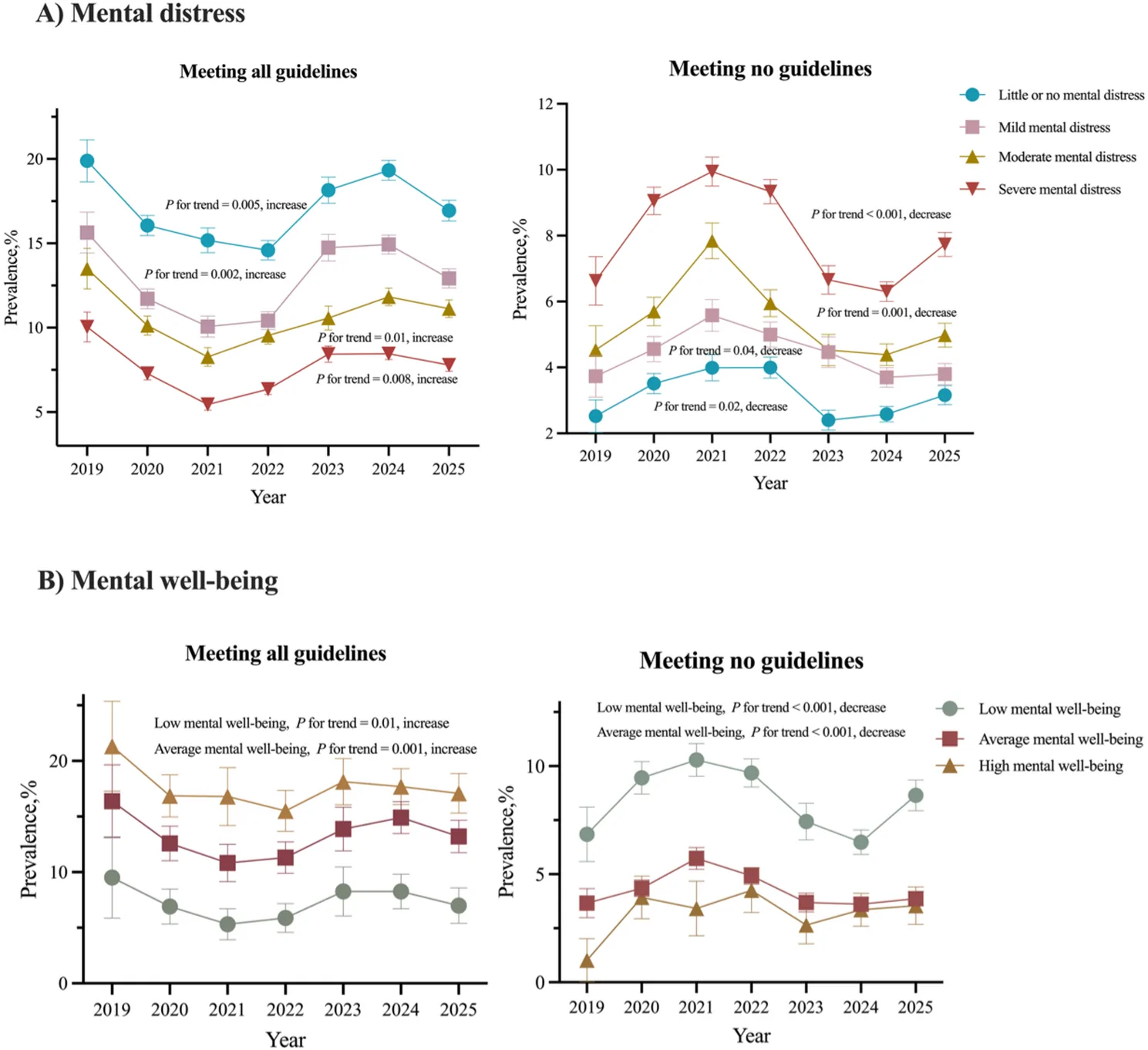
Trends in the prevalence of meeting 24-Hour Movement Guidelines stratified by mental health characteristics, CCWS 2019–2025. **A **Mental distress. **B **Mental well-being

The temporal trends in meeting 24-HMG stratified by mental health characteristics are shown in Fig. 4.

#### Mental distress

The prevalence of meeting all guidelines followed a clear gradient by distress severity. Participants experiencing severe mental distress consistently reported the lowest prevalence of meeting all guidelines, falling from 10.0% in 2019 to a low of 5.5% in 2021 before partially recovering to 7.8% by 2025, and the highest prevalence of meeting none, peaking at 10.0% in 2021 before declining to 7.7% by 2025. Across all distress categories, significant increasing trends in meeting all guidelines (and significant decreasing trends in meeting none were observed (all *p* < 0.05).

#### Mental well-being

A similar gradient was observed for mental well-being. Participants with high mental well-being consistently reported the highest prevalence of meeting all guidelines, declining from 21.3% in 2019 to 17.1% in 2025, and the lowest prevalence of meeting none. Significant increasing trends in meeting all guidelines were observed among those with low (*p* = 0.01) and average mental well-being (*p* = 0.001), but not among those with high mental well-being. Correspondingly, significant decreasing trends in meeting none were observed in the low (*p* < 0.001) and average (*p* < 0.001) groups, with no significant trend for those with high mental well-being. Stratified prevalence estimates for individual 24-HMG components are reported in eTables 11 and 12.

Multivariable logistic regression results are presented in Table 3. Compared with students who were < 20 years, older age was associated with higher likelihood of meeting all guidelines, with the highest odds observed in participants aged over 35 (OR = 1.90, *p* < 0.001). Non-binary/Two-Spirit participants were less likely to meet all guidelines (*p* < 0.001) and more likely to meet none (*p* < 0.001). Men were less likely to meet none of the guidelines (*p* < 0.001). All racialized groups demonstrated lower odds of meeting all guidelines and higher odds of meeting none compared with White participants (Ors ranging from 0.42 to 0.85, all *p* < 0.05). Higher parental education were more likely to meet all guidelines (all *p* < 0.05). Conversely, compared to those reporting no financial stress, students experiencing a great deal of financial stress reported a lower likelihood of meeting all guidelines (OR = 0.79, *p* < 0.001) and higher likelihood of meeting none (*p* < 0.001). A dose-response relationship was observed: increasing levels of mental distress were associated with a progressively lower likelihood of meeting all guidelines (mild: OR = 0.71; moderate: OR = 0.54; severe: OR = 0.38, all *p* < 0.001) and a higher likelihood of meeting none (mild: OR = 1.38; moderate: OR = 1.74; severe: OR = 2.64, all *p* < 0.001). Similarly, higher mental well-being was strongly associated with meeting all guidelines. Across survey years, participants generally showed a higher likelihood of meeting all guidelines and a lower likelihood of meeting none over time, independent of other covariates. Analyses for individual 24-HMG components demonstrated patterns consistent with the observed temporal trends. After covariate adjustment, the likelihood of meeting aerobic PA (OR = 1.05, *p* < 0.001) and overall SB (OR = 1.08, *p* < 0.001) recommendations modestly increased across the study period. In contrast, adherence to recreational screen time (OR = 0.99, *p* = 0.004) and sleep duration (OR = 0.99, *p* < 0.001) recommendations showed slight declines over time. Full regression outputs and stratified temporal trend analyses are provided in Supplementary eTables 6-13.

**Table 3:** Multivariable logistic regression of meeting 24-HMG among post-secondary students: sociodemographic and mental health associations

	Meeting all	Meeting none
	aOR (95%CI)^a^	aOR (95%CI) ^a^
Age (ref < 20)		
20–24	1.11 (1.06, 1.16)***	1.07 (1.01, 1.14)*
25–29	1.39 (1.31, 1.47)***	1.02 (0.94, 1.11)
30–34	1.54 (1.43, 1.66)***	1.03 (0.92,1.15)
Over 35	1.90 (1.78, 2.02)***	0.90 (0.82, 1.00)*
Gender (ref Women)		
Men	0.99 (0.95, 1.03)	0.88 (0.83, 0.93)***
Non-binary/Two-spirit	0.64 (0.56, 0.73)***	1.32 (1.15, 1.52)**
Ethnicity (ref White)		
Black	0.42 (0.37, 0.48)***	1.94 (1.74, 2.17)***
Asian	0.61 (0.58, 0.63)***	1.61 (1.52, 1.70)***
Multiracial	0.76 (0.71, 0.83)***	1.12 (1.00, 1.25)*
Other	0.80 (0.74, 0.86)***	1.41 (1.28, 1.56)**
Parental educational level (ref High school or less)		
College program	1.10 (1.04, 1.16)**	0.90 (0.84, 0.97)*
University degree	1.12 (1.06, 1.18)***	0.91 (0.85, 0.97)**
Graduate or higher	1.22 (1.15, 1.28)***	0.83 (0.77, 0.89)***
Financial stress (ref No financial stress)		
Very little financial stress	1.05 (0.99, 1.13)	1.00 (0.91, 1.10)
Some financial stress	1.06 (1.00, 1.12)	1.01 (0.92, 1.10)
Quite a bit of financial stress	0.97 (0.91, 1.04)	1.07 (0.98, 1.18)
Great deal of financial stress	0.81 (0.75, 0.86)***	1.34 (1.22, 1.47)***
Mental distress (ref Little/No mental distress)		
Mild mental distress	0.71 (0.68, 0.75)***	1.38 (1.26, 1.51)***
Moderate mental distress	0.57 (0.55, 0.61)***	1.73 (1.59, 1.89)***
Severe mental distress	0.39 (0.37, 0.41)***	2.66 (2.47, 2.87)***
Mental well-being (ref Low mental well-being)		
Average mental well-being	2.01 (1.92, 2.10)***	0.47 (0.45, 0.50)***
High mental well-being	2.79 (2.61, 2.98)***	0.38 (0.33, 0.42)***
Year (Continuous)	1.03 (1.02, 1.04)***	0.95 (0.93, 0.96)***
*p* for trend	< 0.001	< 0.001

*Abbreviations: aOR* adjusted Odds Ratio, *CI* Confidence Interval

^a^ Model adjusted for age, gender, ethnicity, parental education, financial stress, mental distress, mental well-being, and survey year

*, *P* < 0.05; **, *P* < 0.01; ***, *P* < 0.001

## Discussion

In this large sample of Canadian post-secondary students, divergent temporal patterns in movement behaviors were observed between 2019 and 2025. Meeting aerobic PA and SB guidelines significantly increased over time, whereas meeting sleep and recreational ST guidelines declined. Although the proportion meeting none of the guidelines decreased significantly, the increase in meeting all components appeared to coincide with the fluctuations observed during the peak pandemic years. Persistent disparities across age, gender identity, race/ethnicity, socioeconomic indicators, and mental health status highlight the importance of examining movement components separately and continuously monitoring 24-HMG trajectories to inform focused and equitable health interventions.

A notable finding of this study is the divergence between total SB and recreational ST. While adherence to SB guidelines increased, meeting the recreational ST recommendation declined, which may partly reflect differences in measurement and underscores the importance of a more contextual understanding of SB. This pattern supports the conceptual distinction between total sedentary time and screen-based sedentary behaviors [49]. The improvement in SB aligns with accelerometer-based data from the Canadian Health Measures Survey (2007–2017), which showed a significant decline in daily sedentary time among young adults aged 18–34 years [50]. However, the concurrent decline in meeting recreational ST aligns with 10-year trends in the US [51] and post-pandemic data in Canada [52], where recreational ST has increased and the majority of adults fail to meet recommendation limits. This discrepancy may reflect shifts in how young adults spend their leisure time, driven by technological advances and widespread smartphone use [50]. Unlike other forms of SB, recreational ST is often driven by habit, such as mindless scrolling on smartphones [52]. This prolonged screen use, particularly in the evening, can delay sleep onset and reduce sleep duration [53]. This pattern may be related to the concurrent decline in students meeting the sleep guidelines observed in our study, a trend similarly reported in Chinese [54] and US adult populations [51] over the last decade.

The prevalence of meeting the PA guidelines increased from 2019 to 2025, which aligns with US trends aged ≥ 20 demonstrating a decrease in physical inactivity [51]. Notably, this upward trajectory appears to contrast with earlier Canadian surveillance data, where sufficient aerobic PA among young adults aged 18–24 years decreased from 56.5% in 2011 to 49.7% in 2019 [55]. In contrast to these general population trends, the CCWS specifically captures post-secondary students, whose schedules and campus contexts may differ from the broader adult population. The student population may benefit from flexible academic schedules and access to subsidized campus recreation programming and infrastructure [56]; however, whether these environmental factors contributed to post-pandemic PA recovery in this sample cannot be determined from the present study and warrants further investigation. Additionally, pandemic-related changes in commuting and hybrid learning may have altered discretionary time available for movement behaviors [57]. Emerging evidence suggests that some young adults reported greater engagement in leisure-time MVPA during the post-pandemic period, potentially related to changes in daily schedules and routines [58].

Overall, only 11.39% of students met all 24-HMG components over the 7-year period. While this is slightly higher than a previous Canadian estimate (9.9%) [59], it remains critically low. Studies investigating a 3-component model (excluding recreational ST) generally report much higher prevalence, ranging from 11.7% in ASEAN countries to 26.9% in Central Europe and up to 27.8% in China [16, 59–65]. The sharp drop in prevalence when recreational ST is integrated into the 24-HMG suggests that digital consumption may represent a key behavioral barrier associated with a lower likelihood of achieving a healthy movement profile. This is consistent with data from Nordic countries, where young adults (18–24 years) are highly physically active but simultaneously report the highest levels of recreational screen use (averaging 3.5–4 h/day), potentially associated with lower overall guideline compliance despite strong PA levels [66].

The modest increase in meeting all guidelines observed over the study period should therefore be interpreted with caution. In sensitivity analyses excluding 2020–2021, the overall trend became non-significant. Instead of reflecting steady recovery, the post-pandemic period (2023–2025) suggests a more complex pattern of behavioral change. Although prevalences of meeting PA, SB, and recreational ST guidelines improved, meeting the sleep guideline continued to decline. One possible explanation is that sleep decline may be associated with structural demands of a fully reopened campus environment, such as commuting and early morning classes, rather than reflecting the screen-based patterns that dominated during lockdowns [67]. In addition, while meeting the recreational ST guideline improved relative to the pandemic nadir, it remained lower than the 2019 baseline (34.3%-38.4% vs. 43.5%). This pattern suggests that elevated digital engagement may have persisted beyond the pandemic period [52, 68]. Taken together, these competing behavioral shifts, specifically gains in activity alongside reduced sleep and sustained screen exposure, may help explain the limited improvement in meeting the overall 24-HMG.

Notably, our trend analysis revealed persistent sociodemographic disparities over the 7-year period. Students who were younger (≤ 24 years), non-binary/Two Spirit, or Black consistently demonstrated a lower prevalence of meeting the integrated 24-HMG, which were consistent with previous evidence on the young adults [37, 59, 69, 70]. Regarding age, students under 24 years largely represent undergraduates transitioning from adolescence to emerging adulthood. This developmental stage is characterized by increasing autonomy and shifting routines [26]. Compared with older students, younger individuals may experience greater variability in sleep and screen-use patterns [71, 72]. These factors may contribute to challenges in maintaining balanced time allocation across physical activity, sedentary behavior, screen time, and sleep, making it more difficult to meet all movement guidelines.

Regarding gender, the prevalence of meeting 24-HMG was comparable between men and women and increased over time in both groups. However, non-binary and Two-Spirit students consistently exhibited the lowest prevalence. This pattern may be interpreted in light of minority stress theory, which suggests that sexual and gender minority individuals experience elevated levels of social stressors, including discrimination and exclusion [73]. Such stressors are associated with higher mental health burdens among LGBTQA+ young adults and may pose barriers to health-promoting behaviors, such as participation in organized physical activity and maintaining regular sleep patterns [69]. Similarly, ethnicity disparities persisted over time. Compared with White students, racialized students, particularly Black participants, demonstrated lower integrated adherence. Ethnicity operates as a social determinant of health, and systemic inequities may shape access to resources, safe environments, and supportive contexts that facilitate healthy movement behaviors [74]. Consistent with previous literature, our findings indicated socioeconomic disparities in meeting the 24-HMG [75, 76].

An important finding of the current study was the graded association between mental health and meeting the 24-HMG aligning with previous findings [77]. Students reporting higher mental well-being and lower levels of mental distress were significantly more likely to meet the guidelines [11, 59, 65]. These findings are consistent with prior literature describing bidirectional association between movement behaviors and mental health. While healthier movement patterns have been associated with better mental well-being, poor mental health may also make it more difficult to maintain balanced daily routines.

Collectively, these associations highlight the importance of integrating 24-HMG promotion into campus mental health initiatives. International public health frameworks increasingly emphasize this integration, and in the Canadian context, it aligns with national standards advanced by the Canadian Standards Association [78]. In practice, the persistently low prevalence of meeting all 24-HMG components, driven largely by low adherence to recreational screen time recommendations, suggests that campus health promotion strategies may benefit from moving beyond traditional physical activity messaging to more explicitly address digital consumption behaviors. Persistent disparities across sociodemographic subgroups and mental health status further underscore the importance of equity-informed, subgroup-tailored programming. From a surveillance perspective, future studies should continue monitoring integrated movement behavior profiles rather than isolated components to better capture behavioral trends and health inequities within post-secondary populations over time.

### Strengths and limitations

This study has several strengths. To our knowledge, this is the first study to examine temporal trends in meeting 24-HMG among Canadian post-secondary students across multiple years (2019–2025), incorporating a broad set of sociodemographic and mental health indicators to extend understanding of meeting 24-HMG from a broader population health perspective.

Several limitations, however, must be acknowledged. First, the CCWS uses an institutional opt-in approach rather than probability sampling; although not nationally representative, the sample spans 97 Canadian post-secondary institutions with demographic characteristics broadly comparable to national enrollment distributions. Second, the repeated cross-sectional design supports temporal surveillance but precludes causal inference and assessment of intra-individual behavioral change. Approximately 17–23% of responses were excluded because of missing data; however, sensitivity analyses suggested that this was unlikely to meaningfully influence observed trend trajectories. Third, the PA measure captured general aerobic activity but did not include muscle-strengthening activities or separately quantify active travel, both of which may contribute to overall movement behaviors. Fourth, sedentary behavior and recreational screen time were assessed using single-item self-report measures. As with all self-reported measures, they are subject to recall and social desirability biases. The use of single-item measures may limit reliability and validity when assessing these complex behaviors. The survey also did not distinguish between specific recreational screen time contexts (e.g., cognitively active vs. passive use), which may relate differently to health outcomes [20]. The 3-hour threshold for adult recreational screen time remain debated [79], suggesting that our persistently low prevalence of meeting overall 24-HMG may partly reflect this stringent criterion. Additionally, data collection was restricted to Fall and Winter academic terms and did not include the summer period. Although this approach was intended to capture behaviors during the regular academic year, movement behaviors may vary across seasons, and residual seasonal effects cannot be ruled out. Furthermore, part of the Winter 2020 data collection period overlapped with the initial COVID-19-related campus closures and transition to remote instruction in Canada, which may have influenced students’ movement behaviors and may have contributed to observed temporal patterns. Finally, intersectional analyses (e.g., gender × ethnicity) were not feasible because annual subgroup sizes were insufficient for stable trend estimation. Future studies using larger pooled samples, targeted oversampling, and more context-specific assessment of recreational screen time are warranted.

## Conclusions

In this national study of Canadian post-secondary students from 2019 to 2025, the prevalence of meeting none of the 24-HMG significantly declined. Recreational screen time appears to represent the primary behavioral barrier to overall adherence, consistently showing the lowest prevalence and a declining trend across the seven-year period. Persistent disparities across age, gender, and ethnicity suggest that current health inequities are not resolving naturally. Campus-based and public health interventions may need to move beyond generic health promotion approaches and more explicitly address digital habits, screen time reduction, and the integration of mental health support. Continuous, intersectional population surveillance remains essential to track these evolving behavioral trajectories and inform equitable health policies in the digital era.

## Supplementary Information

Supplementary Material 1.

Supplementary Material 2.

## Data Availability

The datasets analyzed during the current study are available through the Canadian Campus Wellbeing Survey (CCWS) website at [https://www.ccws-becc.ca/].

## Abbreviations

24-HMG: 24-Hour Movement Guidelines
PA: Physical activity
MVPA: Moderate-to-vigorous physical activity
SB: Sedentary behavior
ST: Screen time
US: United States
CCWS: Canadian Campus Wellbeing Survey
IPAQ: International Physical Activity Questionnaire
ISAT: International Sedentary Assessment Tool
CI: Confidence intervals
OR: Odds ratio
VIF: Variance Inflation Factor
