# Supplementary material for: Meeting 24-hour movement guidelines among Canadian post-secondary students: temporal trends, disparities, and mental health associations
Source: Int J Behav Nutr Phys Act. 2026 Jul 16;23:96. doi: 10.1186/s12966-026-01952-7 (PMC13678986; doi:10.1186/s12966-026-01952-7)
Supplement: Supplementary file 1 [file 12966_2026_1952_MOESM1_ESM.docx]

**Supplementary materials**

[eFigure1. Sociodemographic stability across survey years, CCWS 2019-2025	2](#_Toc231807825)

[eTable 1. Detailed information of questionnaire items among 24-hour movement guidelines	3](#_Toc231807826)

[eTable 2. Comparison of sociodemographic and mental health characteristics between participants included in the analytic sample and those excluded due to missing data	4](#_Toc231807827)

[eTable 3. Distribution of sociodemographic characteristics among canadian post-secondary students, n (%), CCWS 2019-2025	5](#_Toc231807828)

[eTable 4. Sample sizes and prevalence in meeting 24-hour movement guidelines among Canadian post-secondary Students	6](#_Toc231807829)

[eTable 5. Sensitivity analysis of trends excluding peak COVID-19 years (2020-2021)	6](#_Toc231807830)

[eTable 6. Prevalence (%) and trends in meeting 24-hour movement guidelines stratified by age group, CCWS 2019 - 2025	6](#_Toc231807831)

[eTable 7. Prevalence (%) and trends in meeting 24-hour movement guidelines stratified by gender group, CCWS 2019 - 2025	7](#_Toc231807832)

[eTable 8. Prevalence (%) and trends in meeting 24-hour movement guidelines stratified by ethnic group, CCWS 2019 - 2025.	8](#_Toc231807833)

[eTable 9. Prevalence (%) and trends in meeting 24-hour movement guidelines stratified by parental educational level, CCWS 2019 - 2025	8](#_Toc231807834)

[eTable 10. Prevalence (%) and trends in neeting 24-hour movement guidelines stratified by financial stress, CCWS 2019 - 2025	9](#_Toc231807835)

[eTable 11. Prevalence (%) and trends in meeting 24-hour movement guidelines stratified by mental distress, CCWS 2019 - 2025	10](#_Toc231807836)

[eTable 12. Prevalence (%) and trends in meeting 24-hour movement guidelines stratified by mental well-being, CCWS 2019 - 2025	11](#_Toc231807837)

[eTable 13. Multivariable logistic regression associations between sociodemographic and mental health characteristics and meeting the 24-hour movement guidelines among Canadian post-secondary students, CCWS 2019-2025	12](#_Toc231807838)

eFigure1. Sociodemographic stability across survey years, CCWS 2019-2025

eTable 1. Detailed information of questionnaire items among 24-hour movement guidelines

| Movement behaviors | Questions | Response |
| --- | --- | --- |
| Physical activity | (i)Vigorous PA: “*During the last 7 days, on how many days did you do vigorous physical activities like heavy lifting, running, or fast bicycling?*” and (ii) Moderate PA: “*During the last 7 days, on how many days did you do moderate physical activities like carrying light loads, bicycling at a regular pace or brisk walking?*” | 0-7 days  specific times (e.g., 2 hours 30 minutes) |
| Sedentary behavior | *“During the last 7 days, how much time did you usually spend sitting during the full day? Include time spent at school or work, at home, while doing course work, and during leisure time. This may include time spent sitting at a desk, visiting friends, reading or sitting or lying down to watch television.”* | 0-24 hours per day  0-59 minutes per day |
| Recreational screen time | *“On a typical weekday in the past week, how much time did you spend (i) Sitting, reclining, or lying down and watching TV or using a computer, tablet or smartphone during your free time? Include time spent texting, watching DVDs, videos, playing computer games, Xbox, PlayStation, iPod, YouTube, Facebook or other social networking tools, emailing and using the Internet. Do not include time spent on a computer for work or at school”.* | 0-24 hours per day  0-59 minutes per day |
| Sleep | *“During the past week, what time have you usually turned out the light and gone to sleep on weekdays?”*  *“During the past week, what time have you usually woke up in the morning on weekdays?”*  *“During the past week, what time have you usually turned out the light and gone to sleep on weekends?”*  *“During the past week, what time have you usually woke up in the morning on weekends?”* | choose a time between 12PM-11:30AM |

eTable 2. Comparison of sociodemographic and mental health characteristics between participants included in the analytic sample and those excluded due to missing data

| Characteristic | Included (n = 116,597) | Excluded (n = 30,464) | P-value |
| --- | --- | --- | --- |
| Age Group, n (%) |  |  | <0.001 |
| *Under 20* | 31,510 (27.0%) | 7,646 (25.1%) |  |
| *20 - 24* | 50,990 (43.7%) | 12,556 (41.2%) |  |
| *25 - 29* | 15,786 (13.5%) | 4,326 (14.2%) |  |
| *30 - 34* | 7,755 (6.7%) | 2,272 (7.5%) |  |
| *Over 35* | 10,556 (9.1%) | 3,664 (12.0%) |  |
| Gender Identity, n (%) |  |  | <0.001 |
| *Women* | 78,623 (67.4%) | 17,417 (63.9%) |  |
| *Men* | 34,862 (29.9%) | 9,146 (33.6%) |  |
| *Non-binary / Two-Spirit* | 3,112 (2.7%) | 698 (2.6%) |  |
| Race/Ethnicity, n (%) |  |  | <0.001 |
| *White* | 56,397 (48.4%) | 7,999 (31.1%) |  |
| *Asian* | 39,848 (34.2%) | 11,893 (46.2%) |  |
| *Black* | 4,613 (4.0%) | 1,960 (7.6%) |  |
| *Multi-racial* | 7,417 (6.4%) | 1,473 (5.7%) |  |
| *Other* | 8,322 (7.1%) | 2,404 (9.3%) |  |
| Parental Education, n (%) |  |  | <0.001 |
| *High School or less* | 23,479 (20.1%) | 5,576 (26.8%) |  |
| *College / Technical* | 24,890 (21.4%) | 4,407 (21.2%) |  |
| *University* | 40,574 (34.8%) | 6,450 (31.0%) |  |
| *Graduate degree* | 27,654 (23.7%) | 4,346 (20.9%) |  |
| Financial Stress, n (%) |  |  | <0.001 |
| *None* | 14,005 (12.0%) | 3,382 (11.4%) |  |
| *Little* | 23,601 (20.2%) | 4,696 (15.8%) |  |
| *Some* | 34,706 (29.8%) | 8,458 (28.5%) |  |
| *Quite a bit* | 23,023 (19.8%) | 5,916 (20.0%) |  |
| *A great deal* | 21,262 (18.2%) | 7,192 (24.3%) |  |
| Psychological Distress (K10), n (%) |  |  | <0.001 |
| *None* | 27,532 (23.6%) | 6,083 (21.4%) |  |
| *Mild* | 24,184 (20.7%) | 5,199 (18.3%) |  |
| *Moderate* | 24,046 (20.6%) | 5,537 (19.4%) |  |
| *Severe* | 40,835 (35.0%) | 11,657 (40.9%) |  |
| Mental Well-being (WEMWBS), n (%) |  |  | <0.001 |
| *Low well-being* | 38,370 (32.9%) | 9,453 (34.5%) |  |
| *Average well-being* | 68,898 (59.1%) | 14,727 (53.7%) |  |
| *High well-being* | 9,329 (8.0%) | 3,229 (11.8%) |  |

eTable 3. Distribution of sociodemographic characteristics among canadian post-secondary students, n (%), CCWS 2019-2025

|  | 2019 | 2020 | 2021 | 2022 | 2023 | 2024 | 2025 | Total |
| --- | --- | --- | --- | --- | --- | --- | --- | --- |
| **Age group** |  |  |  |  |  |  |  |  |
| *Under 20* | 1,365  (27.53) | 5,151  (28.33) | 5,513  (36.74) | 5,879  (27.56) | 2,662  (21.48) | 5,545  (23.06) | 5,395  (26.09) | 31,510  (27.02) |
| *20 - 24* | 2,032  (40.98) | 7,919  (43.56) | 6,014  (41.07) | 9,630  (45.15) | 4,908  (39.61) | 10,606  (44.10) | 9,881  (47.78) | 50,990  (43.73) |
| *25 - 29* | 804  (16.21) | 2,513  (13.82) | 1,817  (12.11) | 2,797  (13.11) | 1,876  (15.14) | 3,319  (13.80) | 2,660  (12.86) | 15,786  (13.54) |
| *30 - 34* | 336  (6.78) | 2,136  (6.25) | 783  (5.22) | 1,315  (6.17) | 1,168  (9.43) | 1,858  (7.73) | 1,159  (5.60) | 7,755  (6.65) |
| *Over 35* | 422  (8.51) | 1,462  (8.04) | 880  (5.86) | 1,708  (8.01) | 1,777  (14.34) | 2,721  (11.31) | 1,586  (7.67) | 10,556  (9.05) |
| **Gender** |  |  |  |  |  |  |  |  |
| *Women* | 32,269  (65.92) | 12,605  (69.33) | 10,492  (69.91) | 14,242  (66.77) | 8,240  (66.50) | 16,322  (67.87) | 13,453  (65.05) | 78,623  (67.43) |
| *Men* | 1,618  (32.63) | 5,307  (29.19) | 4,218  (28.11) | 6,508  (30.51) | 3,717  (30.00) | 6,852  (28.49) | 6,642  (32.12) | 34,862  (29.90) |
| *Non-binary/Two-spirit* | 72  (1.45) | 269  (1.48) | 297  (1.98) | 579  (2.71) | 434  (3.50) | 875  (3.64) | 586  (2.83) | 3,112  (2.67) |
| **Ethnicity** |  |  |  |  |  |  |  |  |
| *White* | 1,801  (36.32) | 9,101  (50.06) | 8,175  (54.47) | 9,001  (42.20) | 7,238  (58.41) | 12,851  (53.44) | 8,230  (39.79) | 56,397  (48.37) |
| *Black* | 59  (1.19) | 444  (2.44) | 456  (3.04) | 873  (4.09) | 512  (4.13) | 998  (4.15) | 1,271  (6.15) | 4,613  (3.96) |
| *Asian* | 2,386  (48.11) | 6,486  (35.67) | 4,550  (30.32) | 8,334  (39.07) | 3,245  (26.19) | 7,229  (30.06) | 7,618  (36.84) | 39,848  (34.18) |
| *Multiracial* | 12  (6.29) | 983  (5.41) | 956  (6.37) | 1,359  (6.37) | 620  (5.00) | 1,448  (6.02) | 1,739  (8.41) | 7,417  (6.36) |
| *Other* | 401  (8.09) | 1,167  (6.42) | 870  (5.80) | 1,762  (8.26) | 776  (6.26) | 1,523  (6.33) | 1,823  (8.81) | 8,322  (7.14) |
| **Parental educational level** |  |  |  |  |  |  |  |  |
| *High school or less* | 1,106  (22.30) | 3,985  (21.92) | 2,597  (17.31) | 4,012  (18.81) | 3,053  (24.64) | 4,656  (19.36) | 4,070  (19.68) | 23,479  (20.14) |
| *College Program* | 904  (18.23) | 4,201  (23.11) | 3,128  (20.84) | 4,282  (20.08) | 3,273  (26.41) | 5,202  (21.63) | 3,900  (18.86) | 24890  (21.35) |
| *University degree* | 1,733  (34.95) | 6,050  (33.28) | 5,314  (35.41) | ,721  (36.20) | 3,848  (31.05) | 8,545  (35.53) | 7,363  (35.60) | 40,574  (34.80) |
| *Graduate or higher* | 1,216  (24.52) | 3,945  (21.70) | 3,968  (26.44) | 5,314  (24.91) | 2,217  (17.89) | 5,646  (23.48) | 5,348  (25.86) | 27,654  (23.72) |
| **Financial stress** |  |  |  |  |  |  |  |  |
| *No stress* | 551  (11.11) | 2,059  (11.33) | 2,240  (14.93) | 2,834  (13.29) | 1,283  (10.35) | 2,287  (9.51) | 2,751  (13.30) | 14,005  (12.01) |
| *Very little stress* | 1,028  (20.73) | 3,710  (20.41) | 3,641  (24.26) | 4,620  (21.66) | 2,14  (17.27) | 4,165  (17.32) | 4,297  (20.78) | 23,601  (20.24) |
| *Some stress* | 1,444  (29.12) | 5,636  (31.00) | 4,683  (31.21) | 6,430  (30.15) | 3,648  (29.44) | 7,066  (29.38) | 5,799  (28.04) | 34,706  (29.77) |
| *Quite a bit of stress* | 962  (19.40) | 3,748  (20.61) | 2,466  (16.43) | 4,012  (18.81) | 2,627  (21.20) | 5,235  (21.77) | 3,973  (19.21) | 23,023  (19.75) |
| *Great deal of stress* | 974  (19.64) | 3,028  (16.65) | 1,977  (13.17) | 3,433  (16.10) | 2,693  (21.73) | 5,296  (22.02) | 3,861  (18.67) | 21,262  (18.24) |
| **Mental distress** |  |  |  |  |  |  |  |  |
| *Little/no mental distress* | 1,307  (26.36) | 4,755  (26.15) | 3,057  (20.37) | 4,730  (22.18) | 3,206  (25.87) | 5,767  (23.98) | 4,710  (22.77) | 27.532  (23.61) |
| *Mild mental distress* | 1,151  (23.21) | 3,818  (21.00) | 2,919  (19.45) | 4,222  (19.79) | 2,529  (20.41) | 5,050  (21.00) | 4,495  (21.73) | 24,184  (20.74) |
| *Moderate mental distress* | 1,037  (20.91) | 3,632  (19.98) | 3,109  (20.72) | 4,236  (19.86) | 2,450  (19.77) | 4,944  (20.56) | 4,638  (22.43) | 24,046  (20.62) |
| *Severe mental distress* | 1,464  (29.52) | 5,976  (32.87) | 5,922  (39.46) | 8,141  (38.17) | 4,206  (33.94) | 8,288  (34.46) | 6,838  (33.06) | 40,835  (35.02) |
| **Mental well-being** |  |  |  |  |  |  |  |  |
| *Low mental well-being* | 1,533  (30.91) | 5,954  (32.75) | 6,065  (40.41) | 7,805  (36.59) | 3,670  (29.62) | 7,366  (30.63) | 5,977  (28.90) | 37,370  (32.91) |
| *Average mental well-being* | 3,032  (61.14) | 10,751  (59.13) | 8,151  (54.31) | 12,042  (56.46) | 7,398  (59.70) | 14,540  (60.46) | 13,984  (62.78) | 68,898  (59.09) |
| *High mental well-being* | 394  (7.95) | 1,476  (8.12) | 791  (5.27) | 1,482  (6.95) | 1,323  (10.68) | 2,143  (8.91) | 1,720  (8.32) | 9,329  (8.00) |

eTable 4. Sample sizes and prevalence in meeting 24-hour movement guidelines among Canadian post-secondary Students

|  | 2019 | 2020 | 2021 | 2022 | 2023 | 2024 | 2025 | Total | Mean prevalence |
| --- | --- | --- | --- | --- | --- | --- | --- | --- | --- |
| PA | 3,359 | 11,733 | 9,610 | 13,637 | 8,429 | 17,066 | 14,244 | 78,078 | 66.96 |
| SB | 2,881 | 9,732 | 6,197 | 10,642 | 7,449 | 14,447 | 12,215 | 63,563 | 54.52 |
| recreational ST | 2,155 | 6,562 | 5,266 | 7,107 | 4,757 | 9,012 | 7,085 | 41,944 | 35.97 |
| Sleep | 3,106 | 11,195 | 9,639 | 13,427 | 7,548 | 14,953 | 12,497 | 72,185 | 61.91 |
| Meeting all | 727 | 2,014 | 1,338 | 2,052 | 1,569 | 3,155 | 2,428 | 13,283 | 11.39 |
| Meeting none | 220 | 1,089 | 1,118 | 1,412 | 581 | 1,075 | 1,080 | 6,575 | 5.64 |

Abbreviations: PA = physical activity; SB = sedentary behavior; ST = screen time

.

eTable 5. Sensitivity analysis of trends excluding peak COVID-19 years (2020-2021)

| 24-HMB guidelines | Original trend | Sensitivity trend | Conclusion |
| --- | --- | --- | --- |
|  | Includes all years | Excludes 2020-2021 |  |
|  | *β* (95% CI) | *β* (95% CI) |  |
| PA | 0.01 (0.009, 0.011)*** | 0.009 (0.007, 0.011) *** | robust |
| SB | 0.02 (0.018, 0.021)*** | 0.01 (0.01, 0.02) *** | robust |
| Recreational ST | -0.004 (-0.006 - 0.003) *** | -0.006 (-0.008, -0.004) *** | robust |
| Sleep duration | -0.003 (-0.004, -0.001) *** | -0.005 (-0.007, -0.003) ** | robust |
| Meeting all guidelines | 0.003 (0.002, 0.003) *** | 0.001 (-0.001, 0.002) | not significant |
| Meeting none | -0.003 (-0.003, 0.002)*** | -0.001 (-0.002, -0.000)* | robust |

Abbreviations: 24-HMG = 24-hour movement guidelines; PA = physical activity; SB = sedentary behavior; ST = screen time. CI = Confidence Interval.

eTable 6. Prevalence (%) and trends in meeting 24-hour movement guidelines stratified by age group, CCWS 2019 - 2025

|  | 2019 | 2020 | 2021 | 2022 | 2023 | 2024 | 2025 | 7-year Linear Trends  *β* (95%CI) |
| --- | --- | --- | --- | --- | --- | --- | --- | --- |
| **Meeting PA guideline** | | |  |  |  |  |  |  |
| Under 20 72.5 67.2 | | | 64.1 | 66.4 | 75.2 | 76.5 | 74.5 | 0.019 (0.016, 0.022)*** |
| 20 - 24 68.5 64.9 | | | 64.7 | 64.6 | 70.4 | 74.2 | 70.2 | 0.015 (-0.013, 0.017)*** |
| 25 - 29 65.3 63.2 | | | 63.5 | 61.5 | 64.2 | 67.2 | 64.7 | 0.005 (0.001, 0.009)* |
| 30 - 34 64.0 61.3 | | | 62.3 | 58.3 | 62.3 | 60.8 | 60.4 | -0.002 (-0.008, 0.004) |
| Over 35 56.2 58.1 | | | 61.4 | 56.2 | 58.3 | 58.7 | 54.9 | -0.003 (-0.008, 0.003) |
| **Meeting SB guideline** | | |  |  |  |  |  |  |
| Under 20 56.9 49.8 | | | 38.9 | 48.1 | 60.0 | 58.3 | 59.0 | 0.026 (0.023, 0.029)*** |
| 20 - 24 57.3 52.6 | | | 40.7 | 49.4 | 58.9 | 59.6 | 57.9 | 0.019 (0.017, 0.022)*** |
| 25 - 29 60.2 57.4 | | | 41.1 | 51.6 | 57.6 | 58.8 | 59.7 | 0.012 (0.008, 0.016)*** |
| 30 - 34 56.0 57.0 | | | 47.6 | 48.8 | 61.6 | 61.6 | 61.0 | 0.018 (0.012, 0.024)*** |
| Over 35 63.3 62.2 | | | 55.2 | 57.3 | 65.2 | 66.1 | 63.9 | 0.011 (0.006, 0.016)*** |
| **Meeting recreational ST guideline** | | |  |  |  |  |  |  |
| Under 20 35.7 33.5 | | | 32.8 | 29.1 | 32.0 | 31.6 | 31.5 | -0.004 (-0.007, -0.0014)** |
| 20 - 24 41.6 32.8 | | | 31.4 | 30.2 | 33.8 | 34.4 | 32.2 | -0.0014 (-0.0036, 0.001) |
| 25 - 29 51.0 38.3 | | | 40.1 | 36.7 | 39.7 | 40.4 | 36.8 | -0.007 (-0.01, -0.003)** |
| 30 - 34 51.5 45.1 | | | 47.6 | 43.2 | 47.9 | 46.7 | 38.3 | -0.009 (-0.02, -0.003)** |
| Over 35 56.6 52.1 | | | 53.3 | 52.2 | 53.0 | 51.5 | 49.0 | -0.007 (-0.01, -0.001)* |
| **Meeting sleep guideline** | | |  |  |  |  |  |  |
| Under 20 60.1 60.1 | | | 63.0 | 61.0 | 59.8 | 61.4 | 59.1 | -0.002 (-0.005, 0.001) |
| 20 - 24 60.7 60.5 | | | 63.8 | 61.6 | 60.3 | 62.4 | 60.2 | -0.001 (-0.003, 0.001) |
| 25 - 29 67.4 63.5 | | | 67.5 | 63.2 | 62.8 | 62.6 | 62.5 | -0.006 (-0.01, -0.002)** |
| 30 - 34 68.5 65.1 | | | 65.8 | 65.1 | 63.1 | 62.8 | 62.6 | -0.008 (-0.02, -0.002)* |
| Over 35 66.4 66.3 | | | 66.7 | 64.7 | 60.9 | 61.9 | 61.2 | -0.01 (-0.02, -0.006)*** |
| **Meeting All Guidelines** | | |  |  |  |  |  |  |
| Under 20 | 11.7 | 9.0 | 7.4 | 7.8 | 11.5 | 11.0 | 11.6 | 0.006 (0.004, 0.008)*** |
| 20 - 24 | 13.5 | 10.0 | 7.8 | 8.9 | 11.6 | 12.4 | 11.1 | 0.004 (0.002, 0.005)*** |
| 25 - 29 | 18.9 | 12.4 | 11.1 | 10.8 | 12.9 | 14.4 | 12.7 | 0.0000 (-0.003, 0.003) |
| 30 - 34 | 15.8 | 15.1 | 13.0 | 12.0 | 14.3 | 15.9 | 12.2 | -0.001 (-0.005, 0.003) |
| Over 35 | 20.9 | 18.6 | 18.4 | 16.1 | 16.0 | 16.9 | 14.3 | -0.007 (-0.011, -0.003)** |
| **Meeting None** | |  |  |  |  |  |  |  |
| Under 20 | 4.6 | 6.2 | 7.2 | 6.5 | 3.9 | 3.9 | 4.8 | -0.004 (-0.006, -0.003)*** |
| 20 - 24 | 5.0 | 6.4 | 8.2 | 7.0 | 5.1 | 4.3 | 5.2 | -0.004 (-0.005, -0.003)*** |
| 25 - 29 | 2.3 | 5.3 | 7.4 | 6.4 | 5.1 | 5.3 | 5.5 | 0.0002 (-0.002, 0.002) |
| 30 - 34 | 4.2 | 5.2 | 7.4 | 6.5 | 4.6 | 5.1 | 6.3 | 0.0000 (-0.003, 0.003) |
| Over 35 | 4.0 | 4.9 | 4.6 | 5.6 | 4.3 | 5.2 | 5.4 | 0.001 (-0.003, 0.003) |

Abbreviations: PA = physical activity; SB = sedentary behavior; Recreational ST = recreational screen time. *, *P* < 0.05; **, *P* < 0.01; ***, *P* < 0.001.

eTable 7. Prevalence (%) and trends in meeting 24-hour movement guidelines stratified by gender group, CCWS 2019 - 2025

|  | 2019 | 2020 | 2021 | 2022 | 2023 | 2024 | 2025 | 7-year Linear Trends  *β* (95%CI) |
| --- | --- | --- | --- | --- | --- | --- | --- | --- |
| **Meeting PA guideline** | | |  |  |  |  |  |  |
| Women 66.2 62.6 | | | 63.4 | 62.4 | 65.8 | 69.5 | 66.7 | 0.01 (0.009, 0.012)*** |
| Men 70.9 69.2 | | | 66.5 | 67.7 | 73.1 | 74.8 | 73.1 | 0.012 (0.009, 0.014)*** |
| Non-binary/Two-Spirit 68.1 62.8 | | | 52.2 | 59.4 | 67.1 | 67.8 | 70.6 | 0.024 (0.014, 0.034)*** |
| **Meeting SB guideline** | | |  |  |  |  |  |  |
| Women 58.8 54.4 | | | 42.3 | 51.1 | 60.9 | 61.6 | 60.0 | 0.021 (0.019, 0.023)*** |
| Men 56.9 51.9 | | | 39.7 | 48.1 | 60.0 | 58.2 | 58.3 | 0.02 (0.017, 0.023)*** |
| Non-binary/Two-Spirit 51.4 42.0 | | | 29.6 | 40.8 | 44.9 | 46.4 | 46.6 | 0.017 (0.007, 0.028)** |
| **Meeting recreational ST guideline** | | |  |  |  |  |  |  |
| Women 45.7 37.0 | | | 36.3 | 34.5 | 39.5 | 38.6 | 34.9 | -0.003 (-0.005, -0.002)** |
| Men 38.5 34.3 | | | 33.0 | 31.3 | 36.8 | 36.0 | 33.2 | -0.0001 (-0.003, 0.003) |
| Non-binary/Two-Spirit 52.8 28.6 | | | 21.5 | 26.9 | 30.4 | 28.5 | 30.5 | 0.0004 (-0.009, 0.01) |
| **Meeting sleep guideline** | | |  |  |  |  |  |  |
| Women 62.1 60.6 | | | 62.9 | 61.4 | 59.6 | 61.6 | 59.7 | -0.003 (-0.004, -0.001)** |
| Men 63.9 64.0 | | | 68.1 | 64.0 | 63.8 | 63.8 | 62.1 | -0.005 (-0.008, -0.002)** |
| Non-binary/Two-Spirit 58.3 58.4 | | | 56.9 | 58.9 | 60.4 | 59.3 | 59.0 | 0.003 (-0.008, 0.01) |
| **Meeting All Guidelines** | | |  |  |  |  |  |  |
| Woman | 15.0 | 11.2 | 9.1 | 9.9 | 12.5 | 13.4 | 11.6 | 0.003 (0.002, 0.004)*** |
| Man | 14.0 | 10.9 | 8.8 | 9.1 | 13.6 | 13.2 | 12.2 | 0.004 (0.002, 0.006)*** |
| Non-binary/Two-Spirit | 15.3 | 7.4 | 3.4 | 7.6 | 8.9 | 7.0 | 9.7 | 0.003 (-0.003, 0.009) |
| **Meeting None** | |  |  |  |  |  |  |  |
| Woman | 4.5 | 6.2 | 7.6 | 6.8 | 4.7 | 4.6 | 5.5 | -0.003 (-0.004, -0.002)*** |
| Man | 5.1 | 5.6 | 6.7 | 6.0 | 4.5 | 4.1 | 4.6 | -0.003 (-0.004, -0.002)*** |
| Non-binary/Two-Spirit | 6.9 | 7.1 | 14.8 | 8.5 | 6.9 | 5.8 | 6.1 | -0.009 (-0.01, -0.003)** |

Abbreviations: PA = physical activity; SB = sedentary behavior; Recreational ST = recreational screen time.*, *P* < 0.05; **, *P* < 0.01; ***, *P* < 0.001.

eTable 8. Prevalence (%) and trends in meeting 24-hour movement guidelines stratified by ethnic group, CCWS 2019 - 2025.

|  | 2019 | 2020 | 2021 | 2022 | 2023 | 2024 | 2025 | 7-year Linear Trends  *β* (95%CI) |
| --- | --- | --- | --- | --- | --- | --- | --- | --- |
| **Meeting PA guideline** | | |  |  |  |  |  |  |
| White 74.5 69.5 | | | 70.5 | 71.3 | 71.6 | 76.6 | 76.2 | 0.013 (0.011, 0.015)*** |
| Black 67.8 55.0 | | | 49.1 | 56.9 | 61.1 | 61.3 | 62.1 | 0.018 (0.010, 0.026)*** |
| Asian 62.2 58.3 | | | 53.9 | 57.7 | 61.5 | 61.7 | 62.7 | 0.009 (0.006, 0.011)*** |
| Multiracial 74.0 68.1 | | | 65.5 | 65.3 | 70.2 | 75.7 | 73.4 | 0.014 (0.008, 0.019)*** |
| Other 65.3 61.1 | | | 62.9 | 58.1 | 64.6 | 69.0 | 62.0 | 0.006 (0.0002, 0.011)* |
| **Meeting SB guideline** | | |  |  |  |  |  |  |
| White 55.3 53.1 | | | 42.1 | 50.1 | 58.9 | 59.3 | 59.4 | 0.022 (0.020, 0.025)*** |
| Black 66.1 55.6 | | | 44.1 | 52.9 | 62.5 | 64.9 | 63.5 | 0.029 (0.020, 0.037)*** |
| Asian 59.4 53.9 | | | 39.9 | 49.3 | 62.5 | 60.3 | 57.5 | 0.015 (0.013, 0.018)*** |
| Multiracial 55.1 51.1 | | | 38.1 | 46.9 | 56.1 | 58.6 | 59.2 | 0.026 (0.020, 0.032)*** |
| Other 63.8 56.5 | | | 43.2 | 52.1 | 62.9 | 63.6 | 60.8 | 0.017 (0.011, 0.022)*** |
| **Meeting recreational ST guideline** | | |  |  |  |  |  |  |
| White 51.2 38.7 | | | 38.0 | 36.6 | 40.0 | 39.9 | 38.3 | -0.002 (-0.005, 0.0000) |
| Black 44.1 27.3 | | | 25.2 | 23.1 | 28.7 | 28.3 | 27.1 | 0.002 (-0.006, 0.009) |
| Asian 37.3 33.1 | | | 30.7 | 31.2 | 36.6 | 33.6 | 30.8 | -0.003 (-0.005, -0.001)* |
| Multiracial 42.3 36.1 | | | 34.8 | 28.7 | 36.3 | 37.6 | 34.3 | -0.001 (-0.007, 0.005) |
| Other 45.9 35.6 | | | 35.7 | 35.3 | 38.8 | 41.1 | 35.1 | -0.002 (-0.007, 0.004) |
| **Meeting sleep guideline** | | |  |  |  |  |  |  |
| White 68.9 63.2 | | | 65.5 | 65.3 | 63.7 | 65.5 | 63.9 | -0.001 (-0.003, 0.002) |
| Black 52.5 51.6 | | | 56.1 | 53.0 | 45.7 | 47.1 | 49.8 | -0.01 (-0.02, -0.002)* |
| Asian 57.9 60.5 | | | 62.6 | 60.4 | 57.5 | 58.3 | 59.1 | -0.004 (-0.006, -0.001)** |
| Multiracial 61.9 63.3 | | | 66.0 | 63.4 | 58.1 | 63.8 | 60.2 | -0.006 (-0.01, 0.0000) |
| Other 64.8 57.3 | | | 63.3 | 57.7 | 61.5 | 60.7 | 57.9 | -0.003 (-0.008, 0.003) |
| **Meeting All Guidelines** | | |  |  |  |  |  |  |
| White | 20.3 | 13.1 | 10.7 | 12.4 | 14.2 | 15.1 | 14.8 | 0.004 (0.002, 0.005)*** |
| Black | 10.2 | 5.6 | 5.0 | 4.7 | 5.1 | 7.6 | 7.3 | 0.005 (0.001, 0.008)* |
| Asian | 1.7 | 9.1 | 6.5 | 7.3 | 10.4 | 9.8 | 9.4 | 0.002 (0.001, 0.003)* |
| Multiracial | 12.8 | 11.5 | 7.7 | 8.2 | 11.0 | 13.1 | 12.0 | 0.005 (0.001, 0.008)* |
| Other | 15.0 | 8.2 | 8.1 | 9.8 | 13.9 | 15.5 | 10.6 | 0.006 (0.002, 0.009)** |
| **Meeting None** | |  |  |  |  |  |  |  |
| White | 3.9 | 5.0 | 6.3 | 5.0 | 4.0 | 3.5 | 3.7 | -0.004 (-0.005, -0.003)*** |
| Black | 3.4 | 9.7 | 10.8 | 9.5 | 7.0 | 8.1 | 7.1 | -0.005 (-0.01, -0.001)* |
| Asian | 5.0 | 7.1 | 9.9 | 7.7 | 5.7 | 5.9 | 6.6 | -0.002 (-0.003, -0.001)** |
| Multiracial | 4.8 | 4.7 | 5.8 | 6.7 | 5.8 | 3.5 | 4.4 | -0.003 (-0.005, 0.0000) |
| Other | 3.0 | 7.7 | 6.1 | 8.1 | 4.6 | 4.8 | 6.0 | -0.002 (-0.005, 0.001) |

Abbreviations: PA = physical activity; SB = sedentary behavior; Recreational ST = recreational screen time.*, *P* < 0.05; **, *P* < 0.01; ***, *P* < 0.001.

eTable 9. Prevalence (%) and trends in meeting 24-hour movement guidelines stratified by parental educational level, CCWS 2019 - 2025

|  | 2019 | 2020 | 2021 | 2022 | 2023 | 2024 | 2025 | 7-year Linear Trends  *β* (95%CI) |
| --- | --- | --- | --- | --- | --- | --- | --- | --- |
| **Meeting PA guideline** | | |  |  |  |  |  |  |
| High school or less 62.7 61.2 | | | 60.4 | 59.4 | 65.1 | 66.2 | 65.3 | 0.01 (0.007, 0.014)*** |
| College Program 68.5 64.9 | | | 63.7 | 64.1 | 68.1 | 71.6 | 68.4 | 0.011 (0.008, 0.014)*** |
| University degree 68.3 64.3 | | | 64.7 | 64.4 | 68.6 | 71.5 | 69.9 | 0.013 (0.01, 0.016)*** |
| Graduate or higher 70.9 67.9 | | | 65.8 | 66.5 | 70.8 | 73.5 | 70.6 | 0.009 (0.006, 0.012)*** |
| **Meeting SB guideline** | | |  |  |  |  |  |  |
| High school or less 61.0 56.2 | | | 46.1 | 53.6 | 64.1 | 61.5 | 62.3 | 0.017 (0.014, 0.020)*** |
| College Program 59.2 55.9 | | | 42.5 | 52.9 | 62.7 | 60.6 | 61.7 | 0.020 (0.017, 0.023)*** |
| University degree 58.0 51.9 | | | 40.7 | 48.9 | 57.4 | 60.4 | 58.5 | 0.022 (0.019, 0.025)*** |
| Graduate or higher 54.7 50.8 | | | 38.0 | 46.1 | 55.4 | 57.9 | 55.4 | 0.021 (0.017, 0.024)*** |
| **Meeting recreational ST guideline** | | |  |  |  |  |  |  |
| High school or less 41.0 34.2 | | | 34.7 | 31.1 | 36.5 | 34.9 | 31.3 | -0.005 (-0.009, -0.002)** |
| College Program 39.7 34.8 | | | 32.6 | 31.8 | 38.5 | 35.9 | 32.5 | -0.0008 (-0.004, 0.002) |
| University degree 43.6 36.5 | | | 35.4 | 33.5 | 38.4 | 37.8 | 34.4 | -0.003 (-0.006, -0.0004)* |
| Graduate or higher 48.4 38.8 | | | 37.0 | 35.9 | 40.8 | 40.6 | 37.6 | -0.002 (-0.005, 0.0009) |
| **Meeting sleep guideline** | | |  |  |  |  |  |  |
| High school or less 62.2 59.1 | | | 60.1 | 58.3 | 59.3 | 59.2 | 56.5 | -0.005 (-0.008, -0.002)** |
| College Program 61.0 61.1 | | | 61.8 | 62.1 | 60.4 | 61.9 | 59.8 | -0.002 (-0.005, 0.002) |
| University degree 63.1 62.4 | | | 65.9 | 62.2 | 61.7 | 62.6 | 61.1 | -0.004 (-0.007, -0.001)** |
| Graduate or higher 63.7 63.3 | | | 66.6 | 64.8 | 62.6 | 64.3 | 63.0 | -0.002 (-0.005, 0.001) |
| **Meeting All Guidelines** | | |  |  |  |  |  |  |
| High school or less | 12.9 | 9.9 | 9.1 | 8.6 | 12.6 | 11.1 | 10.2 | 0.001 (-0.001, 0.003) |
| College Program | 12.9 | 11.2 | 8.5 | 10.4 | 12.5 | 12.6 | 11.3 | 0.003 (0.001, 0.005)* |
| University degree | 15.5 | 10.8 | 9.0 | 9.3 | 12.7 | 13.4 | 12.1 | 0.004 (0.002, 0.005)*** |
| Graduate or higher | 16.3 | 12.7 | 9.0 | 10.2 | 13.1 | 14.8 | 12.8 | 0.004 (0.001, 0.006)** |
| **Meeting None** | |  |  |  |  |  |  |  |
| High school or less | 4.3 | 6.5 | 8.2 | 7.2 | 5.0 | 5.3 | 5.9 | -0.002 (-0.004, -0.0003)* |
| College Program | 5.5 | 5.9 | 7.9 | 7.0 | 4.5 | 4.2 | 4.9 | -0.004 (-0.006, -0.003)*** |
| University degree | 4.0 | 6.1 | 7.2 | 6.7 | 5.0 | 4.5 | 5.1 | -0.003 (-0.004, -0.002)*** |
| Graduate or higher | 4.4 | 5.5 | 6.9 | 5.7 | 4.0 | 4.0 | 5.1 | -0.002 (-0.004, -0.001)** |

Abbreviations: PA = physical activity; SB = sedentary behavior; Recreational ST = recreational screen time. *, *P* < 0.05; **, *P* < 0.01; ***, *P* < 0.001.

eTable 10. Prevalence (%) and trends in neeting 24-hour movement guidelines stratified by financial stress, CCWS 2019 - 2025

|  | 2019 | 2020 | 2021 | 2022 | 2023 | 2024 | 2025 | 7-year Linear Trends  *β* (95%CI) |
| --- | --- | --- | --- | --- | --- | --- | --- | --- |
| **Meeting PA guideline** | | |  |  |  |  |  |  |
| No stress 66.4 63.3 | | | 64.4 | 63.2 | 67.5 | 70.3 | 69.2 | 0.013 (0.008, 0.017)*** |
| Very little stress 71.7 65.2 | | | 64.1 | 64.2 | 70.5 | 73.1 | 71.4 | 0.014 (0.011, 0.017)*** |
| Some stress 71.3 66.6 | | | 64.6 | 65.3 | 68.1 | 72.4 | 70.4 | 0.01 (0.007, 0.013)*** |
| Quite a bit of stress66.0 63.5 | | | 63.5 | 63.5 | 68.3 | 71.2 | 67.3 | 0.012 (0.009, 0.015)*** |
| Great deal of stress60.7 61.9 | | | 62.9 | 62.3 | 66.0 | 67.4 | 65.2 | 0.01 (0.007, 0.014)*** |
| **Meeting SB guideline** | | |  |  |  |  |  |  |
| No stress 51.9 50.6 | | | 39.2 | 47.1 | 56.4 | 55.4 | 56.1 | 0.021 (0.016, 0.025)*** |
| Very little stress 57.3 51.9 | | | 39.7 | 46.9 | 56.8 | 58.5 | 57.2 | 0.020 (0.016, 0.023)*** |
| Some stress 58.0 53.6 | | | 41.8 | 50.2 | 59.8 | 60.0 | 60.2 | 0.021 (0.018, 0.024)*** |
| Quite a bit of stress60.6 54.9 | | | 42.9 | 52.8 | 62.8 | 62.3 | 60.3 | 0.019 (0.016, 0.022)*** |
| Great deal of stress60.2 55.6 | | | 43.3 | 52.3 | 62.3 | 61.2 | 60.3 | 0.017 (0.013, 0.020)*** |
| **Meeting recreational ST guideline** | | |  |  |  |  |  |  |
| No stress 44.3 37.3 | | | 35.7 | 33.9 | 40.1 | 37.0 | 36.0 | -0.003 (-0.007, 0.002) |
| Very little stress 42.9 36.7 | | | 37.2 | 33.9 | 40.0 | 39.6 | 35.5 | -0.001 (-0.004, 0.002) |
| Some stress 45.8 37.4 | | | 36.1 | 34.1 | 39.2 | 39.6 | 33.9 | -0.004 (-0.007, -0.001)** |
| Quite a bit of stress41.9 35.3 | | | 32.4 | 42.5 | 38.0 | 36.0 | 34.0 | -0.001 (-0.005, 0.002) |
| Great deal of stress41.7 33.1 | | | 31.5 | 31.4 | 35.5 | 34.6 | 32.5 | -0.002 (-0.005, 0.002) |
| **Meeting sleep guideline** | | |  |  |  |  |  |  |
| No stress 66.2 65.6 | | | 67.8 | 66.5 | 64.9 | 67.6 | 65.2 | -0.001 (-0.005, 0.003) |
| Very little stress 66.7 64.8 | | | 66.9 | 65.3 | 65.2 | 66.1 | 63.8 | -0.003 (-0.006, 0.001) |
| Some stress 62.9 62.8 | | | 64.7 | 62.3 | 62.7 | 63.3 | 61.4 | -0.002 (-0.005, 0.001) |
| Quite a bit of stress61.9 59.9 | | | 62.6 | 59.8 | 58.6 | 60.7 | 59.0 | -0.003 (-0.006, 0.001) |
| Great deal of stres 56.7 54.8 | | | 56.2 | 56.4 | 55.4 | 56.7 | 53.4 | -0.002 (-0.006, 0.002) |
| **Meeting All Guidelines** | | |  |  |  |  |  |  |
| No stress | 14.0 | 11.5 | 8.5 | 10.3 | 13.7 | 13.0 | 12.6 | 0.005 (0.002, 0.007)** |
| Very little stress | 15.9 | 11.7 | 9.6 | 9.4 | 14.4 | 14.8 | 12.9 | 0.005 (0.002, 0.007)** |
| Some stress | 16.3 | 12.0 | 9.8 | 10.1 | 13.6 | 14.1 | 12.0 | 0.002 (0.0003, 0.004)* |
| Quite a bit of stress | 14.9 | 10.8 | 8.8 | 9.3 | 12.3 | 13.1 | 11.2 | 0003 (0.0004, 0.005)* |
| Great deal of stress | 11.1 | 8.8 | 6.3 | 8.8 | 10.1 | 10.6 | 10.0 | 0.004 (0.001, 0.006)** |
| **Meeting None** | |  |  |  |  |  |  |  |
| No stress | 4.4 | 5.8 | 6.2 | 6.0 | 4.3 | 4.4 | 4.6 | -0.003 (-0.005, -0.001)** |
| Very little stress | 4.0 | 5.0 | 7.5 | 6.4 | 3.8 | 4.2 | 4.3 | -0.003 (-0.005, -0.002)*** |
| Some stress | 4.1 | 5.8 | 7.2 | 6.2 | 4.7 | 3.9 | 4.6 | -0.004 (-0.005, -0.002)*** |
| Quite a bit of stress | 3.9 | 6.1 | 7.8 | 6.8 | 5.3 | 4.0 | 5.5 | -0.003 (-0.004, -0.001)** |
| Great deal of stress | 6.1 | 7.5 | 8.9 | 8.1 | 5.1 | 5.9 | 7.3 | -0.002 (-0.004, -0.0004)* |

Abbreviations: PA = physical activity; SB = sedentary behavior; Recreational ST = recreational screen time. *, *P* < 0.05; **, *P* < 0.01; ***, *P* < 0.001.

eTable 11. Prevalence (%) and trends in meeting 24-hour movement guidelines stratified by mental distress, CCWS 2019 - 2025

|  | 2019 | 2020 | 2021 | 2022 | 2023 | 2024 | 2025 | 7-year Linear Trends  *β* (95%CI) |
| --- | --- | --- | --- | --- | --- | --- | --- | --- |
| **Meeting PA guideline** | | |  |  |  |  |  |  |
| Little/no mental distress 71.2 69.7 | | | 70.0 | 68.1 | 70.8 | 74.1 | 72.4 | 0.007 (0.004, 0.01)*** |
| Mild mental distress 67.1 65.2 | | | 67.4 | 65.4 | 69.4 | 72.2 | 72.1 | 0.014 (0.011, 0.017)*** |
| Moderate mental distress 69.0 63.8 | | | 63.1 | 66.5 | 67.6 | 70.7 | 69.3 | 0.011 (0.008, 0.014)*** |
| Severe mental distress 64.2 60.4 | | | 59.8 | 59.4 | 65.5 | 68.2 | 64.1 | 0.012 (0.01, 0.015)*** |
| **Meeting SB guideline** | | |  |  |  |  |  |  |
| Little/no mental distress 59.8 60.5 | | | 51.6 | 54.9 | 66.0 | 66.8 | 63.9 | 0.017 (0.014, 0.020)*** |
| Mild mental distress 59.3 55.0 | | | 42.6 | 51.5 | 61.7 | 61.8 | 61.7 | 0.021 (0.018, 0.024)*** |
| Moderate mental distress 58.0 53.2 | | | 40.1 | 49.1 | 58.6 | 58.0 | 58.9 | 0.019 (0.016, 0.022)*** |
| Severe mental distress 55.7 47.3 | | | 36.0 | 46.6 | 55.6 | 55.6 | 54.1 | 0.023 (0.020, 0.025)*** |
| **Meeting recreational ST guideline** | | |  |  |  |  |  |  |
| Little/no mental distress 52.5 43.7 | | | 44.7 | 41.6 | 48.2 | 47.6 | 43.0 | -0.001 (-0.004, 0.003) |
| Mild mental distress 45.7 36.8 | | | 37.2 | 35.3 | 40.7 | 40.6 | 36.9 | 0.001 (-0.003, 0.003) |
| Moderate mental distress 40.3 33.8 | | | 34.9 | 33.1 | 36.0 | 35.3 | 32.1 | -0.004 (-0.007, -0.001)* |
| Severe mental distress 35.9 31.0 | | | 29.2 | 27.6 | 30.9 | 29.8 | 28.0 | -0.005 (-0.007, -0.002)** |
| **Meeting sleep guideline** | | |  |  |  |  |  |  |
| Little/no mental distress 69.1 68.0 | | | 70.8 | 69.0 | 66.7 | 67.6 | 66.6 | -0.004 (-0.007, -0.001)** |
| Mild mental distress 67.3 65.1 | | | 68.7 | 66.5 | 64.7 | 65.8 | 63.7 | -0.004 (-0.008, -0.001)** |
| Moderate mental distress 62.6 60.8 | | | 65.7 | 62.2 | 59.9 | 62.7 | 60.3 | -0.003 (-0.006, 0.0002) |
| Severe mental distress 53.2 54.7 | | | 57.9 | 55.8 | 54.8 | 55.8 | 54.1 | -0.002 (-0.004, 0.001) |
| **Meeting All Guidelines** | | |  |  |  |  |  |  |
| Little/no mental distress | 19.9 | 16.1 | 15.2 | 14.6 | 18.2 | 19.3 | 16.9 | 0.003 (0.001, 0.006)** |
| Mild mental distress | 15.6 | 11.7 | 10.1 | 10.4 | 14.8 | 14.9 | 12.9 | 0.004 (0.002, 0.006)** |
| Moderate mental distress | 13.5 | 10.1 | 8.3 | 9.5 | 10.6 | 11.8 | 11.1 | 0.003 (0.001, 0.005)* |
| Severe mental distress | 10.0 | 7.3 | 5.5 | 6.4 | 8.4 | 8.5 | 7.8 | 0.002 (0.001, 0.004)** |
| **Meeting None** | |  |  |  |  |  |  |  |
| Little/no mental distress | 2.5 | 3.5 | 4.0 | 4.0 | 2.4 | 2.6 | 3.2 | -0.001 (-0.003, -0.0002)* |
| Mild mental distress | 3.7 | 4.6 | 5.6 | 5.0 | 4.5 | 3.7 | 3.8 | -0.002 (-0.003, -0.001)** |
| Moderate mental distress | 4.5 | 5.7 | 7.9 | 6.0 | 4.5 | 4.4 | 5.0 | -0.003 (-0.005, -0.002)** |
| Severe mental distress | 6.6 | 9.1 | 10.0 | 9.3 | 6.7 | 6.3 | 7.7 | -0.005 (-0.006, -0.003)*** |

Abbreviations: PA = physical activity; SB = sedentary behavior; Recreational ST = recreational screen time. *, *P* < 0.05; **, *P* < 0.01; ***, *P* < 0.001.

eTable 12. Prevalence (%) and trends in meeting 24-hour movement guidelines stratified by mental well-being, CCWS 2019 - 2025

|  | 2019 | 2020 | 2021 | 2022 | 2023 | 2024 | 2025 | 7-year Linear Trends  *β* (95%CI) |
| --- | --- | --- | --- | --- | --- | --- | --- | --- |
| **Meeting PA guideline** | | |  |  |  |  |  |  |
| Low mental well-being 62.0 56.9 | | | 56.6 | 56.8 | 62.5 | 65.7 | 61.4 | 0.013 (0.011, 0.016)*** |
| Average mental well-being 69.6 67.8 | | | 68.4 | 67.5 | 70.0 | 73.1 | 71.5 | 0.009 (0.007, 0.011)*** |
| High mental well-being 75.9 71.6 | | | 75.7 | 72.5 | 72.2 | 74.5 | 74.9 | 0.003 (-0.002, 0.008) |
| **Meeting SB guideline** | | |  |  |  |  |  |  |
| Low mental well-being 50.9 45.2 | | | 33.9 | 43.2 | 52.5 | 52.7 | 50.6 | 0.021 (0.018, 0.024)*** |
| Average mental well-being 60.1 56.5 | | | 45.2 | 52.7 | 61.8 | 62.4 | 61.4 | 0.018 (0.016, 0.020)*** |
| High mental well-being 70.6 65.3 | | | 57.6 | 62.8 | 71.7 | 70.0 | 70.8 | 0.014 (0.009, 0.019)*** |
| **Meeting recreational ST guideline** | | |  |  |  |  |  |  |
| Low mental well-being 37.6 31.3 | | | 30.8 | 27.6 | 32.8 | 31.6 | 27.7 | -0.005 (-0.008, -0.003)*** |
| Average mental well-being 45.4 38.0 | | | 37.4 | 36.0 | 39.8 | 39.5 | 36.4 | -0.003 (-0.005, -0.001)** |
| High mental well-being 51.0 41.5 | | | 44.2 | 41.4 | 46.3 | 43.7 | 40.9 | -0.003 (-0.009, 0.002) |
| **Meeting sleep guideline** | | |  |  |  |  |  |  |
| Low mental well-being 55.8 55.8 | | | 59.8 | 57.3 | 54.6 | 56.3 | 55.3 | -0.003 (-0.006, -0.001)* |
| Average mental well-being 65.6 64.3 | | | 67.5 | 65.2 | 63.8 | 65.0 | 63.1 | -0.004 (-0.006, -0.002)** |
| High mental well-being 66.8 65.2 | | | 64.5 | 62.6 | 62.4 | 63.4 | 58.4 | -0.01 (-0.02, -0.005)** |
| **Meeting All Guidelines** | | |  |  |  |  |  |  |
| Low mental well-being | 9.5 | 6.9 | 5.3 | 5.9 | 8.3 | 8.3 | 7.0 | 0.002 (0.0004, 0.003)* |
| Average mental well-being | 16.4 | 12.6 | 10.8 | 11.3 | 13.9 | 14.9 | 13.2 | 0.003 (0.001, 0.004)** |
| High mental well-being | 21.3 | 16.9 | 16.8 | 15.5 | 18.1 | 17.7 | 17.1 | -0.0003 (-0.005, 0.004) |
| **Meeting None** | |  |  |  |  |  |  |  |
| Low mental well-being | 6.9 | 9.5 | 10.3 | 9.7 | 7.4 | 6.5 | 8.7 | -0.004 (-0.005, --0.002)*** |
| Average mental well-being | 3.7 | 4.4 | 5.7 | 4.9 | 3.7 | 3.6 | 3.9 | -0.002 (-0.003, -0.001)*** |
| High mental well-being | 1.0 | 3.9 | 3.4 | 4.3 | 2.7 | 3.4 | 3.6 | 0.0002 (-0.002, 0.002) |

Abbreviations: PA = physical activity; SB = sedentary behavior; Recreational ST = recreational screen time. *, *P* < 0.05; **, *P* < 0.01; ***, *P* < 0.001.

eTable 13. Multivariable logistic regression associations between sociodemographic and mental health characteristics and meeting the 24-hour movement guidelines among Canadian post-secondary students, CCWS 2019-2025

|  | PA | SB | Recreational ST | Sleep |
| --- | --- | --- | --- | --- |
|  | aOR (95%*CI*) | aOR (95%*CI*) | aOR (95%*CI*) | aOR (95%*CI*) |
| **Age** (ref *< 20*) |  |  |  |  |
| *20 - 24* | 0.91 (0.88 - 0.93)*** | 1.09 (1.06 - 1.12)*** | 1.05 (1.01 - 1.08)* | 1.03 (1.00 - 1.06) |
| *25 - 29* | 0.75 (0.72 - 0.78)*** | 1.15 (1.11 - 1.20)*** | 1.38 (1.33 - 1.44)*** | 1.13 (1.09 - 1.18)*** |
| *30 - 34* | 0.65 (0.62 - 0.69)*** | 1.23 (1.17 - 1.30)*** | 1.76 (1.67 - 1.85)*** | 1.15 (1.09 - 1.21)*** |
| *Over 35* | 0.57 (0.54 - 0.60)*** | 1.56 (1.49 - 1.63)*** | 2.31 (2.21 - 2.42)*** | 1.11 (1.06 - 1.16)*** |
| **Gender** (ref *Women*) |  |  |  |  |
| *Men* | 1.30 (1.26 - 1.33)*** | 0.91 (0.89 - 0.94)*** | 0.88 (0.86 - 0.90)*** | 1.14 (1.11 - 1.17)*** |
| *Non-binary/Two-spirit* | 0.98 (0.91 - 1.05) | 0.61 (0.57 - 0.66) | 0.69 (0.63 - 0.74)*** | 0.92 (0.85 - 0.99)** |
| **Ethnicity** (ref *White*) |  |  |  |  |
| *Black* | 0.53 (0.50 - 0.57)*** | 1.22 (1.15 - 1.30)*** | 0.57 (0.53 - 0.61)*** | 0.55 (0.52 -0.58)*** |
| *Asian* | 0.55 (0.53 - 0.56)*** | 1.00 (0.98 -1.03) | 0.76 (0.74 - 0.78)*** | 0.81 (0.78 - 0.83)*** |
| *Multiracial* | 0.88 (0.84 – 0.93)*** | 0.94 (0.89 - 1.03)* | 0.83 (0.79 - 0.87)*** | 0.91 (0.86 - 0.96)** |
| *Other* | 0.63 (0.60 - 0.66)*** | 1.14 (1.09 - 1.19)*** | 0.93 (0.88 – 0.97)* | 0.88 (0.76 - 0.84)*** |
| **Parental educational level** (ref *High school or less*) | | |  |  |
| *College Program* | 1.19 (1.15 - 1.24)*** | 0.94 (0.91 - 0.98)** | 1.02 (0.99 - 1.06) | 1.11 (1.07 - 1.15)*** |
| *University degree* | 1.21 (1.17 - 1.26)*** | 0.84 (0.81 - 0.86)*** | 1.10 (1.06 - 1.14)*** | 1.17 (1.13 - 1.21)*** |
| *Graduate or higher* | 1.32 (1.28 - 1.37)*** | 0.75 (0.73 - 0.78)*** | 1.23 (1.18 - 1.27)*** | 1.25 (1.21 - 1.30)*** |
| **Financial stress** (ref *No stress*) | |  |  |  |
| *Very little stress* | 1.09 (1.04 - 1.14)** | 1.05 (1.01 - 1.10)* | 1.02 (0.98 - 1.07) | 0.96 (0.92 - 1.00) |
| *Some stress* | 1.10 (1.05 - 1.14)*** | 1.18 (1.13 - 1.23)*** | 1.02 (0.98 - 1.06) | 0.86 (0.82 - 0.89)*** |
| *Quite a bit of stress* | 1.01 (0.97 - 1.06) | 1.30 (1.25 - 1.36)*** | 0.93 (0.89 - 0.97)*** | 0.77 (0.73 - 0.80)*** |
| *Great deal of stress* | 0.92 (0.88 - 0.97)** | 1.31 (1.25 - 1.37)*** | 0.88 (0.84 - 0.92)*** | 0.63 (0.61 - 0.66)*** |
| **Mental distress** (ref *Little/No mental distress*) | | |  |  |
| *Mild mental distress* | 0.90 (0.86 - 0.93)*** | 0.83 (0.80 - 0.86)*** | 0.75 (0.73 - 0.78)*** | 0.90 (0.87 - 0.93)*** |
| *Moderate mental distress* | 0.84 (0.81 - 0.87)*** | 0.74 (0.71 - 0.76)*** | 0.64 (0.61 - 0.66)*** | 0.76 (0.74 - 0.79)*** |
| *Severe mental distress* | 0.69 (0.67 - 0.72)*** | 0.62 (0.61 -0.64)*** | 0.51 (0.49 - 0.53)*** | 0.58 (0.57 - 0.60)*** |
| **Mental well-being** (ref *Low mental well-being*) | | |  |  |
| *Average mental well-being* | 1.56 (1.52 - 1.60)*** | 1.57 (1.53 - 1.61)*** | 1.41 (1.37 - 1.45)*** | 1.41 (1.37 - 1.44)*** |
| *High mental well-being* | 1.87 (1.78 - 1.96)*** | 2.41 (2.30 - 2.53)*** | 1.74 (1.66 - 1.83)*** | 1.29 (1.23 - 1.35)*** |
| **Year (Continuous)** | 1.05 (1.04 - 1.06)*** | 1.08 (1.08 - 1.09)*** | 0.99 (0.98 - 0.99)** | 0.99 (0.98 - 0.99)*** |
| ***p* for trend** | < 0.001 | < 0.001 | = 0.004 | < 0.001 |

Abbreviations: PA = physical activity; SB = sedentary behavior; ST = screen time; aOR = adjusted Odds Ratio; CI = Confidence Interval.

^a^ Model adjusted for age, gender, ethnicity, parental education, financial stress, mental distress, mental well-being, and survey year.

*, *P* < 0.05; **, *P* < 0.01; ***, *P* < 0.001.
